# Epigenetic activation of a RAS/MYC axis in H3.3K27M-driven cancer

**DOI:** 10.1038/s41467-020-19972-7

**Published:** 2020-12-04

**Authors:** Sanja Pajovic, Robert Siddaway, Taylor Bridge, Javal Sheth, Patricia Rakopoulos, Byungjin Kim, Scott Ryall, Sameer Agnihotri, Lauren Phillips, Man Yu, Christopher Li, Scott Milos, Palak Patel, Dilakshan Srikanthan, Annie Huang, Cynthia Hawkins

**Affiliations:** 1 Arthur and Sonia Labatt Brain Tumour Research Centre, The Hospital for Sick Children, Toronto, ON Canada; 2 Department of Laboratory Medicine and Pathobiology, University of Toronto, Toronto, ON Canada; 3 Department of Neurological Surgery, Children’s Hospital of Pittsburgh of UPMC, Pittsburgh, PA 15213 United States; 4 Division of Haematology and Oncology, The Hospital for Sick Children, Toronto, ON Canada; 5 Division of Pathology, The Hospital for Sick Children, Toronto, ON Canada

**Keywords:** Cancer models, Paediatric cancer, Mechanisms of disease, Epigenetics, Preclinical research

## Abstract

Histone H3 lysine 27 (H3K27M) mutations represent the canonical oncohistone, occurring frequently in midline gliomas but also identified in haematopoietic malignancies and carcinomas. H3K27M functions, at least in part, through widespread changes in H3K27 trimethylation but its role in tumour initiation remains obscure. To address this, we created a transgenic mouse expressing H3.3K27M in diverse progenitor cell populations. H3.3K27M expression drives tumorigenesis in multiple tissues, which is further enhanced by *Trp53* deletion. We find that H3.3K27M epigenetically activates a transcriptome, enriched for PRC2 and SOX10 targets, that overrides developmental and tissue specificity and is conserved between H3.3K27M-mutant mouse and human tumours. A key feature of the H3K27M transcriptome is activation of a RAS/MYC axis, which we find can be targeted therapeutically in isogenic and primary DIPG cell lines with H3.3K27M mutations, providing an explanation for the common co-occurrence of alterations in these pathways in human H3.3K27M-driven cancer. Taken together, these results show how H3.3K27M-driven transcriptome remodelling promotes tumorigenesis and will be critical for targeting cancers with these mutations.

## Abstract (web summary)

Histone H3 at lysine 27 (H3K27M) is often mutated in cancer but its role in tumour initiation is unclear. Here, the authors generated a transgenic model expressing H3.3K27M from the Fabp7 gene promoter, demonstrating that H3.3K27M can initiate diverse tumorigesis on its own, acting through a RAS/MYC transcriptomic programme.

## Introduction

Diffuse Intrinsic Pontine Glioma (DIPG) is a devastating paediatric high-grade glioma (HGG) that diffusely penetrates the brainstem and is the leading cause of paediatric brain tumour death^1^. Surgery is not possible, radiation is largely palliative, and conventional chemotherapy and targeted agents have proven ineffective. Around 80% of DIPGs harbour lysine 27-methionine (K27M) substitutions in histones H3.3 (*H3F3A*) and H3.1 (*HIST1H3B/C*)^2,3^. More recently H3K27 mutations have also been found in a range of other tumours including haematopoietic malignancies and carcinomas^4–8^. Thus, H3K27M is assumed to be an oncohistone, although the precise nature of its oncogenic function remains obscure.

H3K27 is a key hub for transcriptional regulation: acetylation (H3K27ac) is associated with active enhancers while trimethylation (H3K27me3) represses transcription^9–11^. H3K27M has increased affinity for, and inhibits the activity of, Enhancer of zeste homologue 2 (EZH2), the catalytic subunit of Polycomb repressive complex 2 (PRC2) that deposits H3K27me3, leading to widespread H3K27me3 loss^12,13^. However, H3K27M also leads to H3K27me3 gains in some areas, suggesting that aberrant gene expression and repression may both be important^14^.

Despite clonal analysis indicating that H3K27M is the tumour-initiating mutation in DIPG^15,16^, in vivo models of H3K27M-driven cancer, which to date have focused on brain tumours, have failed to generate tumours without additional oncogenic drivers. The endogenous *H3f3a* locus was modified to allow H3.3K27M expression in Nestin-positive cells, where neonatal H3.3K27M expression decreased the latency of gliomas driven by *Trp53* loss and constitutively active *Pdgfra*^17^. In experiments using RCAS-TVA retroviral delivery, neonatal targeting of Nestin-positive brainstem progenitors with H3.3K27M accelerated tumours driven by *Pdgfb* overexpression and *Trp53* loss^18^ Similarly, H3.3K27M decreased the latency of glioma formation in *in utero* electroporation experiments introducing H3.3K27M alongside *Trp53* loss, *Pdgfb* overexpression, or WT or constitutively active (D842V) *Pdgfra* overexpression^19,20^. None of these models yielded tumours where the sole exogenous mutation was H3.3K27M. Further, to produce tumours, H3.3K27M was introduced alongside other strong oncogenic drivers making it difficult to parse out the direct role of the mutant histone in oncogenesis. Overall, these models suggest that H3K27M cooperates with p53 dysfunction and constitutive RAS/MAPK activation, but is not itself sufficient to initiate tumorigenesis, at least in the cells and developmental time points tested thus far. This cooperation with p53 and the RAS/MAPK pathway is supported by the frequent co-occurrence of *PDGFRA* and *TP53* alterations with H3K27M in human DIPGs, although in human tumours H3K27M is clearly the initiating event^21,22^. Thus, how H3.3K27M alters the cell state to permit or initiate tumorigenesis and the basis of its cooperation with additional mutations remains elusive. A clearer understanding of these mechanisms is critical to developing appropriate therapies for this devastating disease.

Here we used a mouse model expressing H3.3K27M from the *Fabp7* promoter in both neural and non-neural precursor cells to investigate H3K27M-driven cancer. H3.3K27M alone was sufficient to drive development of lymphomas and carcinomas, while *Trp53* loss was required for high grade gliomas to form. H3.3K27M imposed a common transcriptome, mirroring early gliomagenesis, which was shared between H3K27M-driven mouse tumours from all sites, H3.3K27M mouse embryonic brainstem and human DIPGs. A main component of the H3.3K27M transcriptome was an activated RAS/MYC axis which could be targeted therapeutically. H3K27M-mutant tumours acquired secondary mutations to reinforce this activation, suggesting a model where early epigenetic pathway activation by H3K27M is later reinforced by pathway activating genetic mutations and explaining the co-occurrence of these mutations in human DIPG.

## Results

### H3.3K27M increases cancer incidence and leads to early death

CD-1 mice, expressing FLAG/HA-tagged H3.3K27M (*H3f3a-K27M)* under the control of the *Fabp7* promoter, were engineered by microinjection, with colonies established from 3 founders (Supplementary Fig. S1a–c). *Fabp7* promoter-directed H3.3K27M expression was compatible with viable embryonic and postnatal development; adult mice had similar phenotype and litter size to CD1 wild-type mice, although E12.5 and E14.5 H3.3K27M-positive embryos occasionally developed signs of central nervous system haemorrhage (Supplementary Fig. S1d). At the gene expression level, *Fabp7* expression is highest in brain during embryogenesis, but is expressed in adult brain as well as in other embryonic and adult tissues including liver, intestine, heart, lung and bone (Supplementary Fig. S1e). Correspondingly, we saw that H3.3K27M expression was highest in brain, yet readily detectable in other tissues (Supplementary Fig. S1f, g)^23–26^. Importantly, the H3.3K27M-FLAG/HA transgene was not overexpressed versus endogenous H3.3 in the mouse samples or when compared to H3K27M levels in human DIPG (Supplementary Fig. S1h, i).

**Fig. 1:**
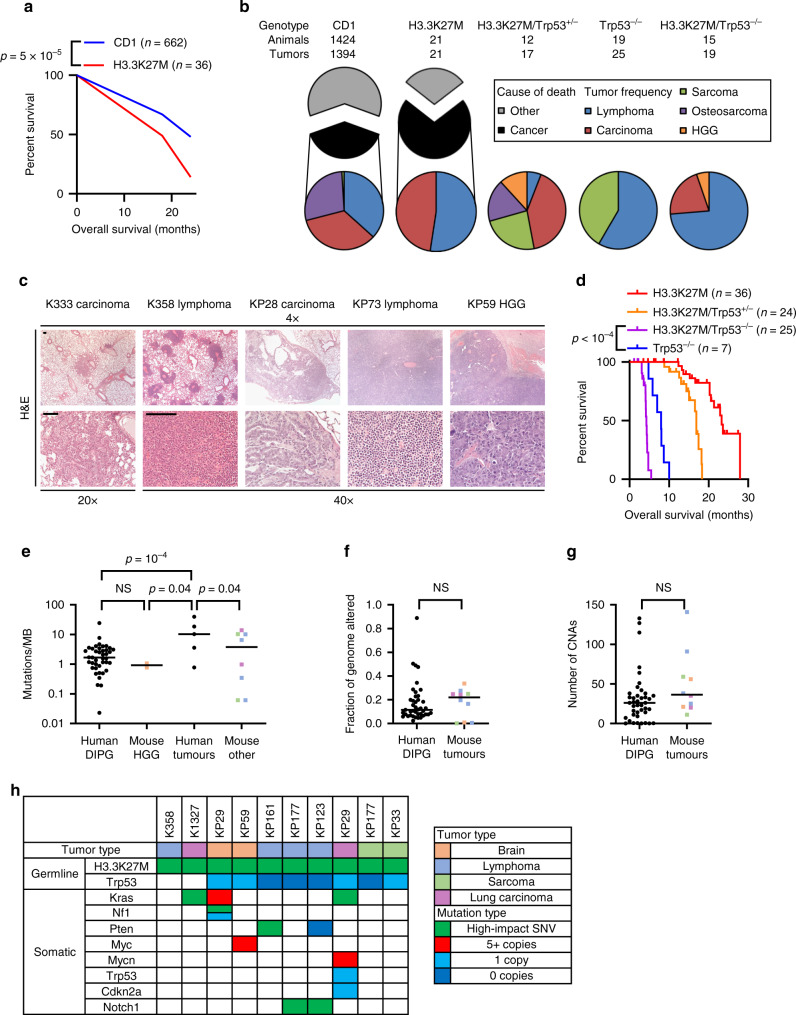
H3.3K27M drives tumorigenesis with genomic changes consistent with DIPG. **a** Survival plot (all-cause mortality) of H3.3K27M mice (red line, *n* = 36) versus CD1 control mice (blue line, *n* = 662)^27^. Significance was determined at 24 months. **b** Pie charts showing the percentage deaths of H3.3K27M and CD1 mice (top, CD1 data from^28–30^) arising from cancer (black) or other causes (grey). The frequency of different tumour types amongst the cancer-related deaths are shown in the coloured pie charts for each genotype where red=carcinoma, blue=lymphoma, purple=osteosarcoma, green=other sarcoma and orange=high grade glioma (HGG) (bottom). **c** Haematoxylin and Eosin (H&E) staining of representative tumours from H3.3K27M mice. Scale bar: 100 µm. **d** Kaplan-Meier survival analysis of H3.3K27M, H3.3K27M/*Trp53*^+/−^, H3.3K27M/*Trp53*^−/−^ and *Trp53*^−/−^ mice^31^. **e** Somatic mutation burden (single-nucleotide variants/indels per megabase (MB) of exome) of human DIPG (*n* = 41), mouse HGG (*n* = 2), and human (*n* = 5) and mouse (*n* = 8) non-gliomas. Mouse tumours are coloured by type as in **h**. **f** Comparison of the fraction of the genome altered between human DIPG (*n* = 41) and mouse (*n* = 10) tumours. Mouse tumours are coloured by type as in **h**. **g** Comparison of copy number alteration (CNA) burden between human DIPG (*n* = 41) and mouse (*n* = 10). Mouse tumours are coloured by type as in **h**. **h** Oncoprint of tumour type, H3.3K27M and *Trp53* genotype plus specific SNVs and focal CNAs. Source data are provided as a Source Data file. Statistical tests: Yates’ Chi-squared (**a**), log rank (**d**), t (**e**, **f**, **g**). NS: Not significant.

H3.3K27M mice had 62% reduced survival (all-cause mortality) compared to CD1 controls at two years (*p* = 5.5 × 10^−5^; Fig. 1a, Supplementary Data 2)^27^, and were 67% more likely to die from cancer (*p* = 0.005; Fig. 1b). They had a 2.7-fold increase in both lymphoma (*p* = 3.1 × 10^−4^) and carcinoma (*p* = 9.6 × 10^−4^) compared with controls (Fig. 1b, Supplementary Data 2)^28–30^. H3.3K27M lymphomas most frequently involved the mediastinum (45%) and were disseminated in 36% of mice, with immunophenotyping showing them to be T-cell lymphomas (B220^−^/CD3^+^; Supplementary Fig. S2a). Carcinomas were usually adenocarcinoma and primarily involved the lung (Fig. 1c), with significantly increased incidence in H3.3K27M mice compared with CD1 controls (*p* = 0.02). Both lymphomas and carcinomas expressed H3.3K27M and FABP7, indicating that they were driven by transgene expression rather than forming spontaneously (Supplementary Fig. S2b). Together, this indicates that H3.3K27M expression drives a significantly increased incidence of lymphoma (*p* = 4.2 × 10^−4^) and carcinoma (*p* = 0.001) in mice. This is the first evidence that H3.3K27M alone can cause cancer and that it functions as a pan-cancer driver.

### H3.3K27M induces gliomas and increases lymphomagenesis in Trp53-deficient mice

As H3.3K27M and *TP53* alterations are highly co-associated in human gliomas, we crossed H3.3K27M mice with *Trp53*^+/−^ deficient animals. Morphologic and immunophenotypic high grade gliomas (HGG) developed at low penetrance in both H3.3K27M/*Trp53*^+/−^ and H3.3K27M/*Trp53*^−/−^ mice, but not in H3.3K27M, *Trp53*^+/−^ or *Trp53*^−/−^ mice (Fig. 1b, c and Supplementary Fig. S2c, Supplementary Data 2). Most had leptomeningeal dissemination, with tumour masses involving the cerebellum, cerebral hemispheres and/or base of the third ventricle. A similar proportion (~70%) of mouse HGG and human DIPG overexpressed *OLIG2* (Supplementary Fig. S2c, d).

H3.3K27M/*Trp53*^−/−^ mice developed more lymphomas (56%) than H3.3K27M (42%) or *Trp53*^−/−^ (32%) animals, and had a 2-fold reduced median survival compared with *Trp53*^−/−^ mice (*p* < 0.0001; Fig. 1b, d, Supplementary Data 2)^31,32^. All tumours in H3.3K27M mice, regardless of tissue of origin, maintained H3.3K27M expression (Supplementary Fig. S2b). Overall, H3.3K27M mice have increased cancer incidence with decreased latency, with gliomas and lymphomas both increased by *Trp53* loss.

### H3.3K27M-driven mouse and human tumours have similar genomic features

We performed whole exome sequencing (WES) on 10 tumours from 8 H3.3K27M, H3.3K27M/*Trp53*^+/−^ or H3.3K27M/*Trp53*^−/−^ mice to investigate the evolution of H3.3K27M-driven tumorigenesis. We compared the genetic features of the H3K27M mutant mouse tumours with those of human brain and non-brain tumours. The mutation frequency was similar between mouse and human tumours (Fig. 1e, Supplementary Data 3)^5,21^. The burden of copy number variations (CNV) and the fraction of the genome affected by CNVs was also similar between mouse tumours and human H3.3K27M DIPG (Fig. 1f, g and Supplementary Fig. S2e; Supplementary Data 3)^21^. Further analysis showed that SNVs and CNVs were acquired in known driver genes of human cancer (including DIPG). These included *Kras* (HGG, carcinoma), *Nf1* (HGG), *Pten* (carcinoma), *Myc* (HGG, carcinoma) and *Notch* (lymphoma) (Fig. 1h). Interestingly, somatic *Trp53* alterations were acquired in 2 tumours, highlighting the cooperative importance of p53 dysfunction in H3.3K27M-driven tumorigenesis. KP29 (H3.3K27M^+/−^) lung carcinoma lost the copy of chromosome 11 (Supplementary Fig. S2e) carrying the wild-type *Trp53* allele, resulting in a *Trp53*^−/−^ tumour, while K1327 (*Trp53*^+/+^) lung carcinoma acquired an R62Q mutation in the p53 transactivation domain, which is critical for apoptotic signalling (Supplementary Fig. S2f)^33^. The latter was detected by Sanger sequencing but with a low mutant allele frequency by WES, indicating that it was a sub-clonal, presumably later-occurring, variant. Overall, H3.3K27M mouse tumours show similar mutation patterns to human DIPG, with frequent mutations in the RAS/MAPK/PI3K pathway (50%) and MYC (20%; Fig. 1h).

### H3.3K27M rapidly disrupts transcriptional networks to establish a glioma-like, proliferative phenotype in mid-development in cooperation with SOX10

In order to investigate the earliest events resulting from H3.3K27M expression we examined brainstem tissue from E14.5 mouse embryos, ~5 days after H3.3K27M expression is induced from the *Fabp7* promoter. We further generated isogenic cell lines expressing H3.3K27M or empty vector (EV) using the human oligodendrocyte precursor cell (OPC) line MO3.13, as OPCs are a proposed cell of origin for DIPG^34^.

**Fig. 2:**
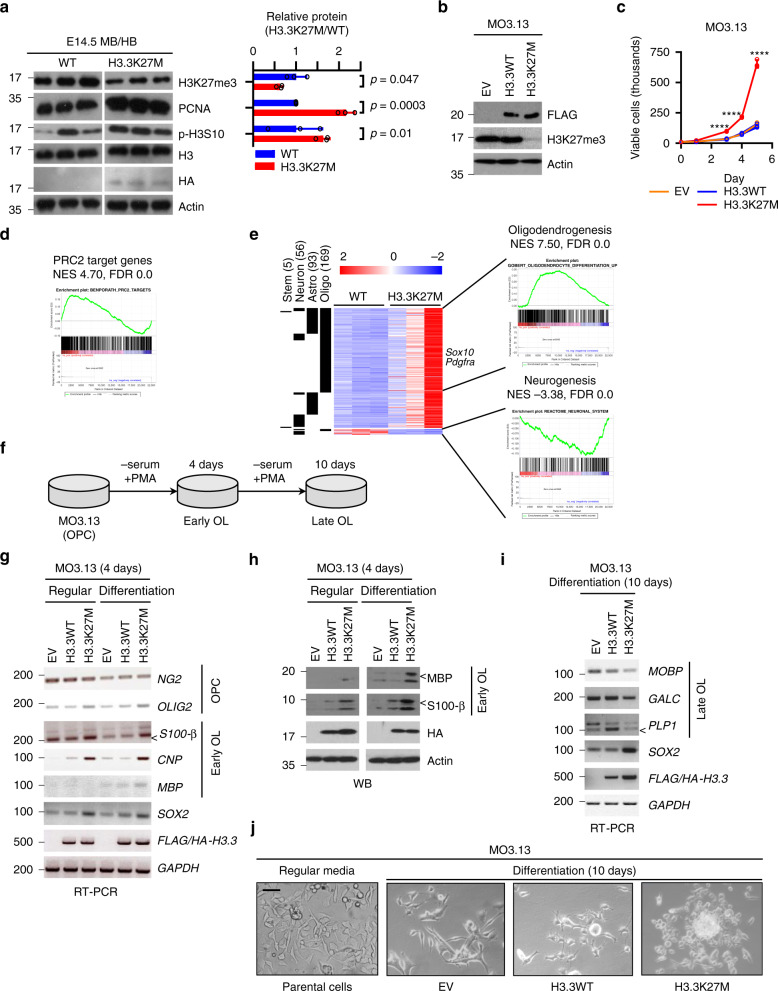
H3.3K27M rapidly induces a glioma-like proliferative phenotype in developing brainstem and subverts oligodendrocyte differentiation programmes. **a** Western blot of H3.3K27M and WT MB/HB from littermates harvested at E14.5. Densitometry quantifies H3.3K27M versus WT (normalised to actin) and is plotted as mean ± standard deviation (*n* = 3 independent embryos). **b** Western blot of empty vector (EV), H3.3WT or H3.3K27M MO3.13 cells (results are representative of 2 independent experiments from different litters). **c** Growth curve of MO3.13 cells transduced with empty vector (EV) or H3.3K27M. Bars show mean ± standard deviation (*n* = 3). **d** GSEA (gene set enrichment analysis) of BENPORATH_PRC2_TARGETS gene set in H3.3K27M E14.5 MB/HB. NES: Normalised Enrichment Score. FDR: false discovery rate. **e** Heatmap and GSEA of differentially expressed genes in H3.3K27M E14.5 MB/HB with functions in different lineages. Oligo: oligodendrocyte, Astro: astrocyte. **f** Schematic of experiment differentiating MO3.13 cells from oligodendrocyte precusor cells (OPC) into early and late oligodendrocytes (OL) experiment using serum starvation and (PMA) phorbol 12-myristate 13-acetate treatment. **g** RT-PCR analysis of MO3.13 cells grown in regular media or differentiated for 4 days. <: specific band. Results are representative of 6 independent replicates. **h** Western blot of MO3.13 cells grown in regular media or differentiated for 4 days. <: specific band. Results are representative of 2 independent replicates. **i** RT-PCR analysis of MO3.13 cells grown in regular media or differentiated for 10 days. <: specific band. Results are representative of 2 independent replicates. **j** Bright-field microscopy images of MO3.13 cells grown in regular media or differentiated for 10 days. Results are representative of 2 independent replicates. Scale bar: 100 µm. Source data are provided as a Source Data file. Statistical tests: t (**a**, **c**). ****p < 0.0001.

H3.3K27M has an inhibitory effect on PRC2, and its expression in E14.5 midbrain/hindbrain (MB/HB) or MO3.13 cells reduced H3K27me3 compared to controls (Fig. 2a, b and Supplementary Fig. S3a). This was accompanied by an increase in the proliferative markers PCNA and phospho-H3S10 in H3.3K27M mice compared with WT (Fig. 2a). A similar increase in proliferation was seen in the MO3.13 cells compared with empty vector (EV) and H3.3WT-expressing control cells (Fig. 2c). In a scratch assay, while EV and H3.3WT cells failed to close a wound after 24 h, H3.3K27M-expressing MO3.13 cells had fully closed the wound, further suggesting that H3.3K27M confers pro-tumorigenic behaviour on MO3.13 cells (Supplementary Fig. S3b).

To better understand the acute transcriptional effects of H3.3K27M expression and the proliferative E14.5 phenotype, we performed RNA sequencing (RNA-Seq), identifying 992 up- and 50 down-regulated genes differentially expressed between H3.3K27M and WT MB/HB at E14.5 (Supplementary Fig. S3c, Supplementary Data 1). H3.3K27M expression level strongly correlated with the degree of gene upregulation (Pearson’s correlation 0.999, *p* = 0.001; Supplementary Fig. S3d, e). In keeping with the known inhibitory effect of H3.3K27M on PRC2, gene set enrichment analysis (GSEA) revealed significantly increased expression of known PRC2 target genes (Fig. 2d), including the PRC2-repressed *Hox* gene family that has important functions in brain development (Supplementary Fig. S3f, g)^35,36^. By chromatin-immunoprecipitation (ChIP) we found that the *Hoxa3*, *Hoxb4* and *Hoxc8* promoters had reduced H3K27me3 in H3.3K27M MB/HB compared with WT (Supplementary Fig. S3h) suggesting that H3.3K27M upregulates them via PRC2 inhibition. H3.3K27M expression in MO3.13 cells also resulted in *Hoxb4* upregulation (Supplementary Fig. S3i).

**Fig. 3:**
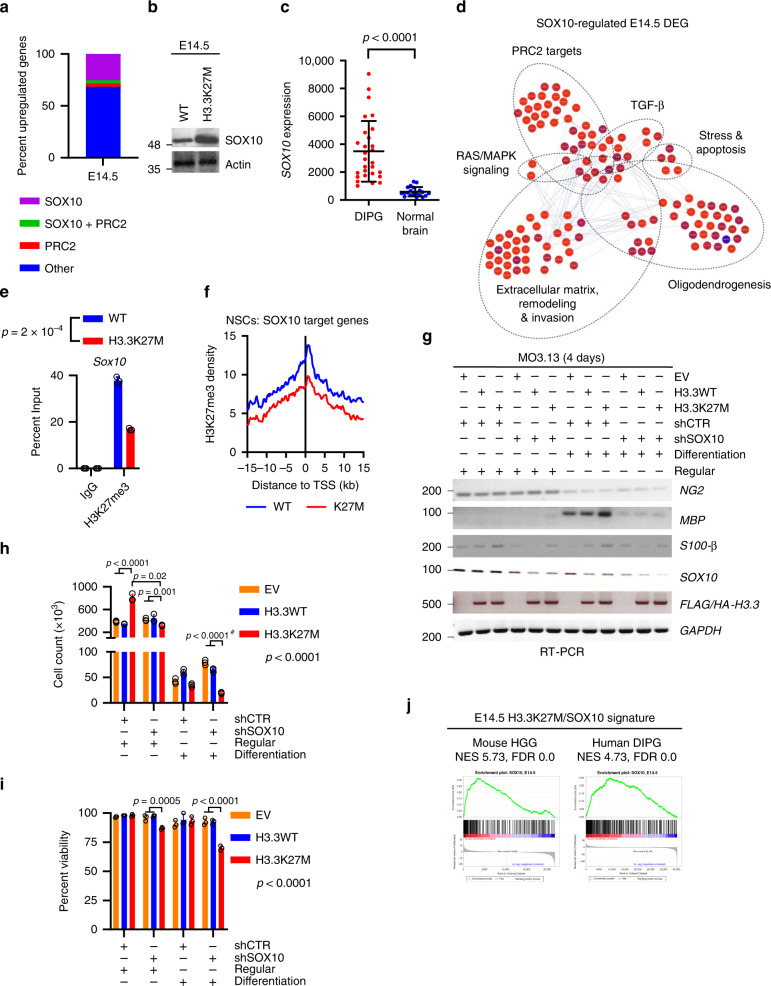
SOX10 is important for the early response to H3.3K27M and activates a signature maintained in gliomas. **a** Percentage of upregulated genes in H3.3K27M E14.5 MB/HB that are directly regulated by PRC2 and/or SOX10. **b** Western blot of H3.3K27M and WT MB/HB from littermates harvested at E14.5. Note that the actin loading control is shared with Fig. 5e (results are representative of at least 3 independent experiments). **c** *SOX10* expression in human H3.3K27M DIPG (*n* = 28) and normal brain (*n* = 20). **d** Network diagram showing SOX10 transcriptional targets from key pathways that are differentially expressed at E14.5. Red: upregulated genes; blue: downregulated genes; colour intensity reflects expression fold-change. DEG: differentially expressed gene. **e** ChIP quantifying H3K27me3 at the *Sox10* promoter in E14.5 WT or H3.3K27M MB/HB. **f** H3K27me3 ChIP-Seq density was profiled ± 15 kb around the (transcription start site) TSS of SOX10 target genes in H3.3K27M or H3.3WT NSCs^46^. **g** RT-PCR analysis of MO3.13 cells transduced with control (shCTR) or SOX10-specific (shSOX10) shRNA and grown for 4 days in regular or differentiation media. Results are representative of 6 independent replicates (shSOX10 clone 1 *n* = 2, clone 2 *n* = 4). EV: empty vector. **h** Cell counts of MO3.13 cells grown as in **g**. Bars show mean ± standard deviation (*n* = 3). **i** Percent viability of MO3.13 cells grown as in **g**. Bars show mean ± standard deviation (*n* = 3). ^#^significance for EV vs H3.3K27M in shSOX10 cells grown in differentiation media was tested by normalising each condition by EV to control for media effect. **j** SOX10 target genes upregulated in H3.3K27M E14.5 MB/HB were used for gene set enrichment analysis (GSEA) in H3.3K27M mouse HGG and human DIPG. Source data are provided as a Source Data file. Statistical tests: t (**c**, **e**), ANOVA (**h**, **i**).

Fewer than 10% of the significantly upregulated genes were direct PRC2 targets as defined in MSigDB^37,38^ indicating that, despite the importance of PRC2 in H3K27M function, pathways beyond PRC2 are also relevant to the transforming effects of H3K27M (Figs. 3a, 5a and Supplementary Fig. S4a). Among the upregulated expressed genes at E14.5, nearly 20% were associated with OPCs/oligodendrocyte differentiation, <10% with astrocyte differentiation and very few with neuronal differentiation or NSCs (Fig. 2e and Supplementary Fig. S4b)^39^. Promoters of neuronal differentiation were downregulated, while several inhibitors of neurogenesis were upregulated (Fig. 2e and Supplementary Fig. S4c)^39^. Oligodendrogenic markers (OLIG1, OLIG2, O4) were increased at both E14.5 and E16.5, neuronal markers (TUJ1, DCBN) were decreased at E14.5 and the astrocyte marker GFAP was unchanged (Supplementary Fig. S4d), suggesting that H3.3K27M affects cell specification.

To evaluate the potential of H3.3K27M to regulate oligodendrocyte transcriptional programmes, we differentiated MO3.13 OPCs into oligodendrocytes (OL) using serum starvation and phorbol 12-myristate 13-acetate (PMA)^40^, as monitored by decreasing *NG2* expression (Fig. 2f, g). S100 calcium binding protein beta (S100-β), myelin basic protein (*MBP*) and 2′,3′-cyclic nucleotide 3′-phosphodiesterase (*CNP*), which are upregulated during OPC differentiation into immature/early OL^41–43^, were induced by H3.3K27M expression and maintained after treatment with differentiation media for 4 days (Fig. 2g, h). *SOX2*, a marker of undifferentiated and cancer stem cells that is increasingly implicated in numerous oncogenic processes^44^, was also induced in H3.3K27M, but not in EV or H3.3WT cells, in both regular and differentiation media (Fig. 2g). This suggested that H3.3K27M may subvert the differentiation process and prompted us to extend differentiation treatment to 10 days (Fig. 2f). The late OL markers myelin associated oligodendrocyte basic protein (*MOBP*), galactosylceramidase (*GALC*) and proteolipid protein 1 (*PLP1*) were expressed in EV and H3.3WT cells with changes in morphology consistent with OL differentiation as compared with MO3.13 cells grown in regular media (Fig. 2i, j). In contrast, H3.3K27M cells were *SOX2*-high, late-OL marker-low, and had a morphology consistent with undifferentiated cells including neurosphere formation (Fig. 2i, j). Taken together, this data implies that H3.3K27M subverts the OPC transcriptome and differentiation programme to promote early/mid OL gene expression but not terminal differentiation, instead continuing to drive proliferation and induce characteristics of undifferentiated cells.

While loss of H3K27me3 is permissive for gene transcription, this loss alone is not enough; transcriptional upregulation is also dependent on the presence of transcription factors. We combined differential gene expression, transcription factor binding sites and protein-protein interactions to identify potential master regulators of the H3K27M phenotype. We found that the transcription factor SOX10 directly regulates ~30% of genes upregulated by H3.3K27M in E14.5 MB/HB, 3.4x more than are direct PRC2-targets (Fig. 3a). SOX10 influences lineage commitment in the developing brain to promote oligodendrogenesis^45^, and we noted that it was upregulated in H3.3K27M MB/HB at E14.5 (Figs. 2e, 3b and Supplementary Fig. S4b, e) as well as H3.3K27M-expressing MO3.13 cells and human H3.3K27M-mutant DIPG (Fig. 3c and Supplementary Fig. S3i), suggesting it may cooperate with H3.3K27M. The genes regulated by SOX10 play key roles in many important biological processes that may be important for mediating the oncogenic function of H3.3K27M (Fig. 3d). A reduction in H3K27me3 at the *Sox10* promoter in H3.3K27M E14.5 MB/HB and throughout the promoter and gene body of H3.3K27M-expressing NSCs^46^ (Fig. 3e and Supplementary Fig. S4f) suggested that H3.3K27M epigenetically mediates *SOX10* upregulation through H3K27me3 loss. Furthermore, H3.3K27M-expresssing NSCs exhibited significant loss of H3K27me3 in the promoters of *Sox10* target genes (Fig. 3f)^46^. Overall, the majority of genes upregulated in the early response to H3.3K27M are not canonical PRC2 target genes. This data suggests that SOX10 may be at least partially responsible for mediating this early response in the MB/HB by promoting oligodendrogenic expansion at the expense of neuronal differentiation.

To better understand how H3.3K27M and SOX10 interact we depleted *SOX10* expression in MO3.13 cells (Supplementary Fig. S4g), and repeated the 4-day differentiation assay (Fig. 2f). *S100-β* and *MBP* are both SOX10 target genes^42^. H3.3K27M did not induce expression of these genes after *SOX10* knockdown, confirming the importance of SOX10 in executing the H3.3K27M transcriptome (Fig. 3g). As expected, all differentiation conditions decreased cell proliferation (*p* < 0.0001; Fig. 3h and Supplementary Fig. S4h). Strikingly, *SOX10* depletion reversed the growth advantage conferred by H3.3K27M in regular media and, when combined with differentiation media, H3.3K27M cells grew substantially more slowly than EV and H3.3WT controls (*p* < 0.0001 when normalising by EV condition to control for the dominating media effect; Fig. 3h and Supplementary Fig. S4h). Moreover, *SOX10* depletion had a synthetic lethal effect with H3.3K27M expression, particularly under differentiation conditions, while EV and H3.3WT cell viability was unaffected (*p* < 0.0001; Fig. 3i and Supplementary Fig. 4i). Collectively these data suggest that H3K27M co-operates with SOX10 in the regulation of transcription programmes involved in cell fate, proliferation, and survival of OL cells consistent with our findings in H3K27M brains, mouse HGGs, and human DIPGs. In keeping with this, the H3.3K27M/SOX10 signature was highly upregulated in both H3.3K27M-mutant mouse HGG and human DIPG (Fig. 3j). Furthermore, 20-30% of differentially expressed genes in the E14.5, mouse HGG and human DIPG datasets are SOX10 target genes, suggesting that SOX10 activity is maintained throughout tumorigenesis.

**Fig. 4:**
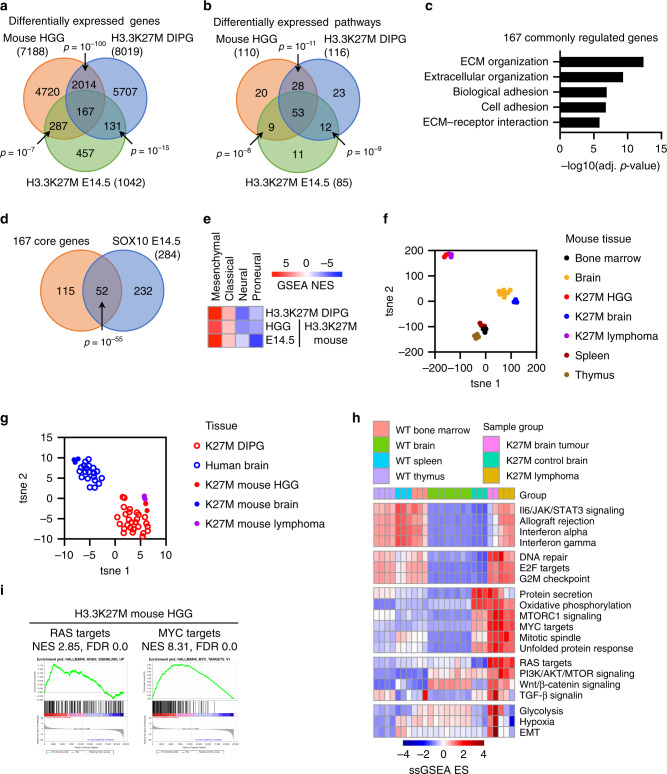
H3.3K27M drives a lineage independent tumour transcriptome. **a** Venn diagram comparing differentially expressed genes in H3.3K27M E14.5 MB/HB and HGG with DIPG. **b** Venn diagram comparing differentially expressed Hallmark and KEGG pathways in H3.3K27M E14.5 MB/HB and HGG with DIPG. **c** Gene ontology analysis of 167 genes commonly regulated in B. **d** Venn diagram showing overlap of 167 genes commonly regulated in B with SOX10 target genes upregulated in E14.5 H3.3K27M MB/HB. **e** Heatmap showing gene set enrichment analysis (GSEA) normalised enrichment scores (NES) of glioblastoma subtype signatures. **f** t-SNE of wild type tissues (bone marrow, spleen, thymus, brain) and untransformed brain, brain tumours and lymphomas from H3.3K27M mice. **g** t-SNE of untransformed brain, brain tumours and lymphomas from H3.3K27M mice and human H3.3K27M DIPG and normal brain. **h** Heatmap of select single sample GSEA (ssGSEA) hallmark enrichment scores (ES). **i** GSEA of RAS and MYC target genes in H3.3K27M mouse HGG. FDR: false discovery rate. Source data are provided as a Source Data file. Statistical tests: Hypergeometric (**a**, **b**, **d**).

To further investigate the similarities between the early effects of H3.3K27M on the brainstem and gliomagenesis we compared the transcriptomes of H3.3K27M mutant E14.5 brainstem with H3.3K27M/Trp53^+/−^ mouse HGGs and human DIPGs. The differentially expressed genes and pathways in all 3 datasets were strikingly similar (all two-way overlaps *p* ≤ 10^−7^; Fig. 4a, b). 167 commonly regulated genes were enriched in extracellular remodelling pathways, consistent with the highly invasive nature of human DIPG (Fig. 4c), and 52 were SOX10 target genes, underscoring the importance of SOX10 activity in establishing the early transcriptional response to H3.3K27M that is maintained throughout tumorigenesis (*p* = 10^−56^, Fig. 4d). The 53 pathways commonly regulated across all 3 datasets included proliferative (RAS signalling, cell cycle, MYC), extracellular remodelling (epithelial-mesenchymal transition, adhesion) and metabolic (glycolysis, oxidative phosphorylation) terms. All 3 datasets showed strong enrichment of the mesenchymal subtype and strong repression of neural/proneural glioblastoma subtype signatures (Fig. 4e)^47^. These data suggest that H3.3K27M rapidly establishes a transcriptome in the brainstem that recapitulates features of gliomagenesis.

### H3.3K27M drives a core transcriptome in different cellular contexts

**Fig. 5:**
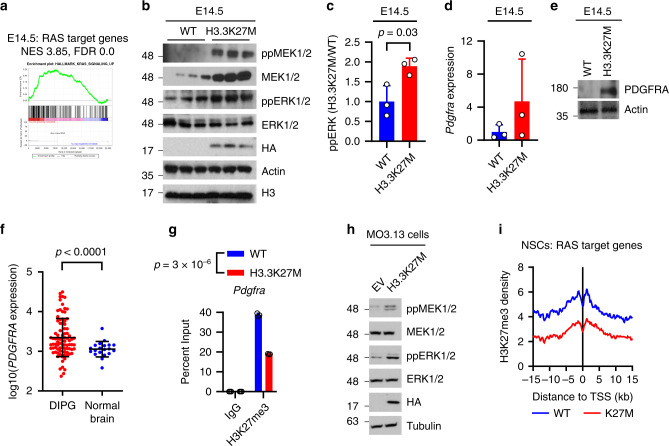
Activation of RAS/MAPK is an early, epigenetically-driven effect of H3.3K27M. **a** Gene set enrichment analysis (GSEA) of RAS target genes in H3.3K27M E14.5 MB/HB. NES: Normalised Enrichment Score. FDR: false discovery rate. **b** Western blot of WT/K27M E14.5 MB/HB (results are representative of 2 independent experiments from different litters). **c** Densitometry quantifying ppERK in B as H3.3K27M vs WT (normalised to actin) and is plotted as mean ± standard deviation (*n* = 3). **d** *Pdgfra* expression was measured by qPCR in 3 independent H3.3K27M or WT embryos, normalised to *Gapdh* and WT, and plotted as mean ± standard deviation. **e** Western blot of H3.3K27M and WT MB/HB from littermates harvested at E14.5. Note that the actin loading control is shared with Fig. 3b (results are representative of at least 3 independent experiments). **f** *PDGFRA* expression in human H3.3K27M DIPG (*n* = 28) and normal brain (*n* = 20). **g** ChIP quantifying H3K27me3 at the *Sox10* promoter in E14.5 WT or H3.3K27M MB/HB. **h** Western blot of empty vector (EV)/H3.3K27M-expressing MO3.13 cells (results are representative of 2 independent experiments). **i** H3K27me3 ChIP-Seq density was profiled ± 15 kb around the transcription start site (TSS) of SOX10 target genes in H3.3K27M or H3.3WT NSCs^46^. Source data are provided as a Source Data file. Statistical tests: t (**c**, **f**, **g**).

To understand the effect of H3.3K27M in different tissues we compared RNA-Seq of HGG, lymphoma and brain from H3.3K27M mice with adult wild-type mouse brain, spleen, thymus and bone marrow^48^, as well as human DIPG and normal brain. Single-sample GSEA (ssGSEA) was used to analyse relative pathway activity across different samples. T-stochastic neighbour embedding (t-SNE) models and hierarchical clustering revealed that, while brain samples clustered separately from bone marrow, spleen and thymus, reflecting expected inter-tissue transcriptional differences, H3.3K27M-driven tumours clustered together, distinctly from their tissues of origin (Fig. 4f and Supplementary Fig. S5a). Similarly, mouse H3.3K27M HGG and lymphomas grouped with human H3.3K27M DIPG, while control H3.3K27M adult mouse brain samples grouped with normal human brain (Fig. 4g). This suggests that H3.3K27M establishes a transcriptome that is conserved across tumour types and overrides tissue-specific expression patterns. Gene sets or transcriptional pathways commonly regulated between H3.3K27M mouse HGG, H3.3K27M mouse lymphoma and human DIPG included RAS, PI3K/AKT signalling and MYC targets (Figs. 4h, 4i, 5a and Supplementary Fig. S5b). Finally, we asked whether the H3.3K27M-SOX10 axis (Fig. 3) has a role in regulation of these pathways. Except for MYC targets at E14.5, which were not upregulated at E14.5 and therefore have no overlap with SOX10-regulated genes at this developmental stage, 30–40% of the RAS/MYC targets and ECM genes differentially expressed in each of H3.3K27M E14.5 MB/HB, mouse HGG and human DIPG are SOX10 target genes (Supplementary Fig. S5c).

Recent data has suggested repression of *CDKN2A* may be important in H3.3K27M-mediated tumorigenesis, as this gene retains its promoter H3K27me3 in the presence of the oncohistone, and the expression level of p16 regulates the proliferative capacity of H3.3K27M-mutant cells^18,46^. We examined *Cdkn2a* promoter H3K27me3 in E14.5 MB/HB, finding that H3.3K27M reduced *Cdkn2a* methylation at this developmental time point (Supplementary Fig. S5d). There was not an accompanying expression change in *Cdkn2a* in RNA-Seq data and, in our mouse HGG and human DIPG datasets, it was upregulated (Supplementary Fig. S5e, f), suggesting that p16 repression is not a key feature of tumorigenesis in this mouse model.

### RAS/MAPK activation is an early response to H3.3K27M

RAS signalling was one of the most strongly activated pathways in established H3.3K27M mouse HGG and lymphoma as well as human DIPG (Fig. 4h, i and Supplementary Fig. S5b). To ask if this was an early response to H3.3K27M, we examined E14.5 MB/HB. At this time point H3.3K27M had been expressed for ~5 days and a proliferative phenotype established (Fig. 2a). Western blotting and RNA-Seq analysis of E14.5 mouse brainstem revealed that RAS/MAPK signalling was indeed already activated in an H3.3K27M associated manner (Fig. 5a–c). *Pdgfra*, one of the main receptors activating RAS signalling, was upregulated in H3.3K27M E14.5 MB/HB, as well as in mouse HGG and human DIPG (Fig. 5d–f and Supplementary Fig. S5h), and the promoters of *Pdgfra*, *Hras*, *Kras* and *Nras* had reduced H3K27me3 at E14.5 (Fig. 5g and Supplementary Fig. S5i). MO3.13 cells expressing H3.3K27M had increased MEK and ERK phosphorylation compared with their empty vector (EV) counterparts (Fig. 5h). H3.3K27M markedly reduced H3K27me3 in the promoters of RAS target genes in NSCs (Fig. 5i)^46^.

**Fig. 6:**
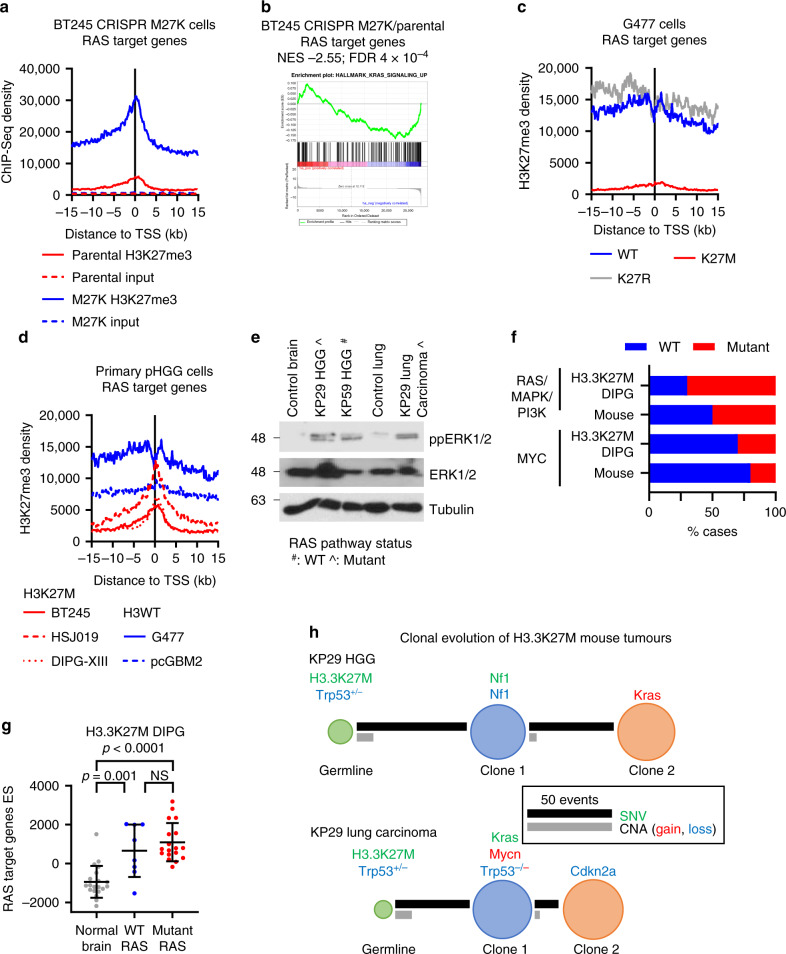
Early epigenetic activation of RAS/MAPK pathway is later reinforced by genetic activation. **a** H3K27me3 ChIP-Seq density was profiled ± 15 kb around the transcription start site (TSS) of RAS target genes in parental and CRISPR-M27K BT245 pHGG cells^49^. **b** Gene set enrichment analysis (GSEA) of RAS target genes in CRISPR-M27K versus parental BT245 pHGG cells^49^. NES: Normalised Enrichment Score. FDR: false discovery rate. **c** H3K27me3 ChIP-Seq density was profiled ± 15 kb around the TSS of RAS target genes in parental, H3.3K27R or H3.3K27M G477 cells^49^. **d** H3K27me3 ChIP-Seq density was profiled ± 15 kb around the TSS of RAS target genes in H3K27M (red) and H3WT (blue) pHGG cells^49^. **e** Western blot of control and H3.3K27M mouse tumour tissue showing increased phospho-ERK in tumours versus their respective normal counterparts (Blots were run twice with similar results). #: RAS pathway WT, ^: RAS pathway mutant. **f** Proportion of H3.3K27M DIPG or mouse tumours with RAS pathway or MYC mutations. **g** RAS target gene ssGSEA ES from RAS pathway WT (*n* = 8)/mutant (*n* = 18) H3.3K27M DIPG and normal brain (*n* = 20). ES: enrichment score. **h** Clonal evolution of single nucleotide (SNV) and copy number (CNV) variants in KP29 high-grade glioma (HGG) and lymphoma, showing known tumour drivers. The circle area of the clones and germline (normal) is proportional to the percentage of the sample comprised of each clone. Note that clone 2 evolved from clone 1, and thus contains all the alterations in clone 1. Driving events are shown. Source data are provided as a Source Data file. Statistical tests: ANOVA (**g**). NS: not significant.

This data suggested that H3.3K27M drives epigenetic activation of the RAS/MAPK pathway by targeting multiple pathway members and its downstream target genes. To further test the dependence of the RAS/MAPK pathway activation on H3.3K27M expression, we next examined H3.3K27M-mutant primary paediatric HGG (pHGG) BT245 cells in which the H3.3K27M mutation had been reversed using CRISPR^49^. BT245-M27K cells showed increased H3K27me3 in the promoters of RAS target genes and significantly downregulated their expression compared with parental H3.3K27M-mutant BT245 cells (Fig. 6a, b). Similarly, depletion of *H3F3A* by shRNA in (*H3F3A*-K27M) H3.3K27M-mutant primary DIPG cells restored RAS target gene promoter H3K27me3 (Supplementary Fig. S6a)^50^. In H3-WT G477 pHGG cells overexpressing H3.3K27M or H3.3K27R^49^, K27R had no effect on H3K27me3 while K27M upregulated RAS target genes and decreased their promoter H3K27me3 (Fig. 6c and Supplementary Fig. S6b). Finally, in a panel of primary pHGG cells, H3.3K27M-mutant cells had reduced RAS target gene promoter H3K27me3 compared with H3WT cells (Fig. 6d)^49^. Together, this suggests that H3.3K27M-mutant tumours exhibit K27M-mediated epigenetic activation of the RAS/MAPK pathway.

### Early epigenetic activation of RAS/MAPK pathway is later reinforced by genetic activation

Based on sequencing data it is well established that mutations in the RAS/MAPK pathway are frequent events in human DIPG^51^ suggesting activation of this pathway is an important step in DIPG tumorigenesis with clonal evolution modelling implicating these as later events^15,16^. Interestingly, our RNAseq data showed activation of the RAS/MAPK pathway in both mouse tumours and human DIPG regardless of whether the RAS/MAPK/PI3K pathway was mutated (Fig. 6e–g, Supplementary Fig. S6c and S6d) suggesting that activation of this pathway may be universal with epigenetic/non-mutation-dependent mechanisms responsible for this activation in the non-mutant cases. Intriguingly, in another mouse model where H3.3K27M accelerated development of *Trp53*^*KO*^*/Pdgfra*-driven HGG^17^, the addition of H3.3K27M epigenetically activated RAS target genes compared to the H3.3WT HGG despite the pathway already being activated by constitutively active PDGFRA (Supplementary Fig. S6e, f)^17^ suggesting epigenetic mechanisms of pathway activation may play a role, even in mutant tumours.

The rapid induction of a RAS/MAPK signalling signature in both our mouse and cell line models following introduction of H3.3K27M as detailed above suggests that epigenetic activation of the pathway may be an early event, with mutation-dependent activation occurring later in tumour development or in a subclonal fashion. This is consistent with a model where the early epigenetic activation of RAS by H3.3K27M is later reinforced by genetic activation of the pathway.

To test if RAS/MAPK genetic alterations in the H3.3K27M mouse tumours are early or late events we investigated the clonal evolution of these tumours (Fig. 6h). For the KP29 HGG and lung carcinoma this also provided a unique opportunity to understand how H3.3K27M affects tumorigenesis of two different tissues within the same animal. These two tumours had little genetic similarity, indicating that both were primary (Fig. 1h and Supplementary Fig. S2e). RAS was activated in both tumours, with *Kras* alterations in the second and first clone of the HGG and lung carcinoma, respectively (Fig. 6h). The HGG additionally had a single copy *Nf1* deletion in clone 1. The lung carcinoma also acquired a focal *Mycn* amplification and single-copy chromosome 11 (*Trp53*) loss in the first clone, and a broad single-copy loss in chromosome 4, containing *Cdkn2a*, in the second (Fig. 6h). Overall, as with human DIPG, most H3.3K27M-driven mouse tumours acquire additional, subclonal, oncogenic alterations beyond the truncal histone mutation and *Trp53* loss. Intriguingly, these alterations converge on the RAS/MAPK/PI3K pathway and MYC/MYCN, which are key features of H3.3K27M DIPG^21^.

### Epigenetic activation of a RAS/MYC axis in H3.3K27M-driven cancer

**Fig. 7:**
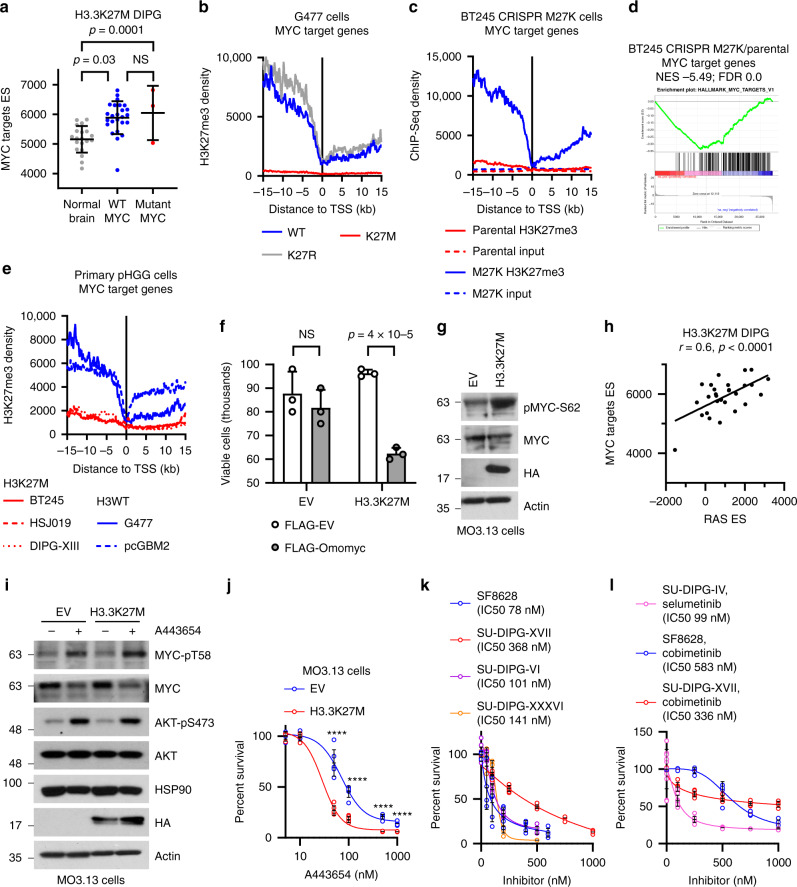
Epigenetic activation of a RAS/MYC axis in H3.3K27M-driven cancers can be targeted therapeutically. **a** MYC target gene single sample gene set enrichment analysis (ssGSEA) enrichment score (ES) from MYC WT (*n* = 25)/mutant (*n* = 3) H3.3K27M DIPG and normal brain (*n* = 20). **b** H3K27me3 ChIP-Seq density was profiled ± 15 kb around the transcription start site (TSS) of MYC target genes in parental, H3.3K27R or H3.3K27M G477 cells^49^. **c** H3K27me3 ChIP-Seq density was profiled ± 15 kb around the TSS of RAS target genes in parental and CRISPR-M27K BT245 pHGG cells^49^. **d** Gene set enrichment analysis (GSEA) of RAS target genes in CRISPR-M27K versus parental BT245 pHGG cells^49^. NES: Normalised Enrichment Score. FDR: false discovery rate. **e** H3K27me3 ChIP-Seq density was profiled ± 15 kb around the TSS of RAS target genes in H3K27M (red) and H3WT (blue) pHGG cells^49^. **f** Viability assay of empty vector (EV) or H3.3K27M MO3.13 cells 3 days post-transfection with FLAG-EV or FLAG-Omomyc plasmids. Bars show mean ± standard deviation (*n* = 3). **g** Western blot of EV and H3.3K27M-expressing MO3.13 cells (results are representative of 2 independent experiments). **h** Scatter plot of ssGSEA ES of RAS and MYC target genes in human H3.3K27M DIPG (*n* = 29). *r*: Pearson correlation. **i** Western blot of EV and H3.3K27M MO3.13 cells treated or not with A443654 (50 nM) for 3 days (results are representative of 2 independent experiments). **j** Viability assay of EV and H3.3K27M MO3.13 cells treated or not with A443654 for 3 days. Bars show mean ± standard deviation (*n* = 4). **k** Viability assays of DIPG cells treated or not with the indicated MEK inhibitor for 3 days. Bars show mean ± standard deviation (*n* ≥ 5). **l** Viability assays of DIPG cells treated or not with A443654 inhibitor for 3 days. Bars show mean ± standard deviation (*n* ≥ 5). Source data are provided as a Source Data file. Statistical tests: ANOVA (**a**, **f**), *t* (**h**, **j**). ****p < 0.0001, NS: not significant.

MYC target genes were among the most upregulated gene sets in H3.3K27M mouse HGG and lymphomas, and human DIPGs (Fig. 4h, i and Supplementary Fig. S5b). As with RAS signalling, H3.3K27M mouse HGG and human DIPG upregulated MYC targets regardless of whether they were *MYC* amplified (Fig. 7a and Supplementary Fig. S7a–c). Overexpression of H3.3K27M in G477 HGG cells or NSCs resulted in loss of H3K27me3 in MYC target genes compared with control cells and their upregulation in H3.3K27M-G477 cells (Fig. 7b and Supplementary Fig. S7d, e)^46,49^. Similarly, the presence of H3.3K27M in *Trp53*^*KO*^*/Pdgfra* mouse HGG led to upregulation of MYC target genes associated with loss of promoter H3K27me3 (Supplementary Fig. S7f, g)^17^. Conversely, depletion of *H3F3A* from H3.3K27M-mutant DIPG cells increased MYC target gene promoter H3K27me3 (Supplementary Fig. S7h)^50^. Furthermore, in BT245 HGG cells, even though *MYC* is amplified in these cells, CRISPR-mediated removal of H3.3K27M resulted in restoration of promoter H3K27me3 and MYC target gene downregulation (Fig. 7c, d)^49^. In agreement with this, primary H3.3K27M-mutant pHGG cells had reduced H3K27me3 at MYC target genes compared with H3WT cells (Fig. 7e)^49^. Together, this data indicates that H3.3K27M drives epigenetic activation of the MYC pathway.

### Therapeutic targeting of RAS/MYC activity in H3.3K27M-driven cancer

There is currently no clinical way to target MYC, but to test whether MYC could be a therapeutic vulnerability in H3.3K27M-mutant cancer, we used Omomyc, a peptide-based dominant negative MYC inhibitor^52^. Omomyc expression led to downregulation of HSP90, a classical MYC target gene, in MO3.13 cells (Supplementary Fig. S7i). Importantly, H3.3K27M MO3.13 cells had significantly increased cell death in response to Omomyc expression compared with control cells (Fig. 7f).

MYC is a known effector gene of RAS signalling, which targets MYC twice (Supplementary Fig. S7j): MYC-S62 phosphorylation by ERK stabilizes MYC and prolongs its activation, and AKT suppresses GSK-3-mediated MYC-T58 phosphorylation leading to MYC degradation^53^. To test if these mechanisms were at play in the context of H3K27M we used H3.3K27M-expressing MO3.13 cells. These had increased phospho-MYC-S62 as well as activated RAS/MAPK compared with their EV counterparts, suggesting that both H3.3K27M and RAS signalling have roles in MYC activation (Figs. 5h, 7g). In support of this, RAS and MYC activation are tightly coupled in H3.3K27M DIPG (Fig. 7h).

We then examined the effects of AKT inhibition on phospho-MYC-T58 in MO3.13 cells. As previously reported, A443654 increased phospho-AKT-S473^54^. Phospho-MYC-T58 was increased in both A443654-treated WT and K27M MO3.13 cells, accompanied by a 30-40% reduction in the overall amount of MYC and HSP90 (Fig. 7i). However, H3.3K27M-MO3.13 cells were significantly more sensitive to A443654 (IC50 29 nM vs EV IC50 72 nM, *p* < 0.0001) than WT MO3.13 cells, suggesting that K27M alone leads to increased dependency on MAPK-mediated MYC activation (Fig. 7j).

Thus, as a clinically viable alternative to directly targeting MYC, targeting either the MAPK or PI3K/AKT arms of the RAS cascade (Supplementary Fig. S7j) could be used. To test this as a potential DIPG therapy we investigated the effects of multiple MEK inhibitors and AKT inhibition on 5 H3K27M-mutant primary DIPG cell lines. Both MEK and AKT inhibition resulted in dose-dependent DIPG cell death with IC50s in the nM range (Fig. 7k, l, Supplementary Fig. S7k, l) supporting these as important therapeutic targets for H3K27M-driven cancer.

## Discussion

H3K27M mutations were first identified as high-frequency drivers of DIPG, and more recently have been found in additional tumour types^2–8^. Previous pre-clinical mouse models of K27M-driven cancer focussed on generating brain tumours and revealed a cooperative effect on p53/PDGFRA-driven mouse HGG^17,19,55^. However, the oncogenic role of H3K27M itself was unclear. To investigate this we created a mouse where H3.3K27M is expressed from the *Fabp7* promoter in radial glia and astrocyte progenitors from E9.5, as well as in developing and adult non-brain tissues^23–26,56^. H3.3K27M mice developed tumours in multiple tissues, with increased frequency and decreased latency, with and without additional *Trp53*-deficiency. Thus, this is the first demonstration that H3.3K27M is sufficient to drive tumorigenesis in the absence of other initiating mutations, establishing its role as an oncohistone. This is in keeping with recent work where removing the K27M mutation from DIPG and HGG cells by CRISPR/Cas9 inhibited their tumorigenicity^49^. HGG occurred exclusively in association with *Trp53* loss in our model, while H3.3K27M alone increased lymphoma and carcinoma susceptibility. Notably, the other tumour types in which H3K27 mutations have been identified in humans are haematopoietic malignancies and carcinomas^6–8^, perhaps explaining the spectrum of H3K27M-related cancers we saw in our model. H3K27M-mediated disruption of PRC2 in these diseases is in keeping with frequent PRC2 deregulation in haematological neoplasms and lung carcinomas, either by mutation (Supplementary Fig. S8) or differential expression^57–61^. Finally, previous studies showed an association of high levels of H3.3 and invasive phenotypes in already developed cancers^62,63^. While our in vitro studies support a phenotypic effect related to the mutant histone, rather than to ectopic expression of H3, our study is limited by the lack of a mouse line expressing the wildtype histone under the Fabp7 promoter. However, transgene expression in our model was very modest compared with endogenous H3.3 and H3.3K27M in human DIPG (Supplementary Fig. S1h, i), and the phenotypes we observed were related to cancer initiation rather than to metastasis, suggesting that they are likely due to the K27M mutation rather than histone overexpression.

Although H3.3K27M expression disrupted canonical PRC2 target genes in the E14.5 MB/HB, many other genes were also upregulated, indicating that although PRC2 is important, the mutant histone must also act through additional signalling and transcriptional pathways to drive transformation. SOX10 and its target genes were epigenetically activated by H3.3K27M and, consistent with the role of SOX10 in promoting OPC commitment^45,64,65^, we found increased expression of oligodendroglial markers at the expense of other lineages. Importantly, an OPC-like cell is hypothesised as a putative DIPG cell of origin^34,55^, suggesting that SOX10 may have a key role in mediating the effects of H3.3K27M. This was confirmed in our cell culture model of OPC differentiation where H3K27M and SOX10 cooperated in activation of a mid-differentiation oligodendroglial transcription programme. Inhibition of SOX10 in this model also led to decreased proliferation and survival specifically in H3K27M cells, suggesting that H3K27M may use SOX10 as a proxy for maintaining oligodendroglial-like cells in a proliferative state. Given the frequent tissue specificity of transcription factors, it is likely that H3.3K27M will partner with other transcription factors in non-glial tissues.

H3.3K27M drove tumorigenesis in multiple tissues and, unexpectedly, the transcriptomes of H3.3K27M-driven tumours were far more similar to one another than to other WT tissues, including their tissues of origin. Remarkably, when comparing H3.3K27M mouse tissues with human DIPGs and normal brain, the mouse HGGs and lymphomas grouped with DIPG. Thus, oncohistone-mediated epigenetic changes override tissue-specific programmes to impose a core, lineage-independent H3.3K27M oncotranscriptome.

Among the most enriched pathways between H3.3K27M-driven mouse cancers and human DIPGs were target genes of RAS/MAPK and MYC. We found that, both in vitro and in vivo, these pathways were epigenetically activated in an H3.3K27M-dependent fashion, including through upregulation of *PDGFRA* and *MYC*. This is supported by recent work showing that H3.3K27M leads to in vitro activation of the RAS/MAPK cascade^66^ and the finding that a *MYC* superenhancer was among the most highly activated by the presence of H3K27M^67^. Together, this suggests that H3.3K27M-mediated epigenetic changes activate RAS/MAPK and MYC, with H3.3K27M epigenetically activating multiple members of the RAS/MAPK cascade as well as its downstream transcriptional targets. Notably, similar findings have been made in malignant peripheral nerve sheath tumours, where mutations of the SUZ12 component of the PRC2 complex result in transcriptional activation of RAS/MAPK target genes through chromatin landscape changes^68^.

Interestingly, human DIPG and H3.3K27M-driven mouse tumours both develop secondary RAS/MAPK/PI3K pathway and *MYC* mutations. This suggests a model in which H3.3K27M initiates tumorigenesis in part through epigenetic activation of RAS and MYC and later mutational events act to lock in this activation in a portion of tumours, providing an explanation for the frequent association of *PDGFRA*/*PI3K* and *MYC* alterations in H3.3K27M DIPG (Supplementary Fig. S9).

Importantly, we show here for the first time that H3.3K27M alone can drive tumorigenesis. In addition to offering insight into the function of PRC2 in tumour initiation, our data suggest that H3.3K27M drives cancer by extensive transcriptome remodelling, centred on epigenetic activation of a RAS/MYC axis. Targeting these pathways was synthetically lethal with H3.3K27M providing a potential therapeutic option for treating H3K27M-driven cancers.

## Methods

### Ethical approval and patient samples

The Hospital for Sick Children Animal Care Committee reviewed and approved all procedures conducted on animals, which were performed in compliance with the Animals for Research Act of Ontario and the Guidelines of the Canadian Council on Animal Care. Patient material was collected from patients presenting at The Hospital for Sick Children with a diagnosis of DIPG (median age 7.1 years, 60% male, Supplementary Data 3) and after receiving informed consent. Ethics oversight was provided by the Hospital for Sick Children Research Ethics Board (#1000055059).

### Transgenic construct and mice

*H3f3a* mouse cDNA with C-terminal FLAG/HA tag was cloned into pCAT3-Basic, downstream from the 1.6 kb *Fabp7* promoter, and the gel purified transgene introduced into ICR-CD1 mouse embryos by pronuclear microinjection. Of 11 positive animals, 3 male founders were chosen based on expression to develop colonies. For developmental studies littermates were used where possible, or else stage-matched CD1 control animals. B6.129S2-*Trp53*^tm1Tyj^/J mice were a gift from Dr. Rebecca Gladdy (Mt. Sinai Hospital, Toronto).

Animals were housed at The Hospital for Sick Children animal facility which operates with a 14/10 h light/dark pattern, at 45–55% humidity and within a temperature range of 22–26 °C.

### Cell culture and lentivirus transduction

All cells were regularly confirmed free of mycoplasm. MO3.13 (Cedarlane) and HEK293T cells were maintained in DMEM (VWR) and 10% FBS (Wisent). DIPG cells were maintained in equal ratios of Neurobasal-A and DMEM/F12 media (Invitrogen) supplemented with 10 mM HEPES, 1 mM sodium pyruvate, 100 µM NEAA, 1 × GlutaMAX-I, 1 × antibiotic/antimycotic, 1 × vitamin A-free B-27 supplement (all from Invitrogen); 20 ng/ml EGF, 20 ng/ml FGF-basic 154, 10 ng/ml PDGF-AA, 10 ng/ml PDGF-BB (all from Shenandoah Biotech); 2 µg/ml heparin (StemCell Technologies). SU-DIPG-IV, SU-DIPG-XIII and SU-DIPG-XVII cells were a generous gift from Michele Monje (Stanford University). SF8628 DIPG cells were from Millipore. pCDH-FLAG/HA expression constructs were generated by inserting H3.3K27M cDNA between the XbaI/BamHI sites of pCDH-CMV-MCS-EF1α-copGFP (SystemBioscience) that had previously had a FLAG/HA tag inserted between the BamHI/NotI sites. Control (EV) or H3.3K27M plasmids were packaged into lentivirus by co-transfecting HEK293T cells with psPAX2 (Addgene#12260) and pMD2.G (Addgene#12259) using Lipofectamine 2000 (Invitrogen). SOX10 shRNA (TRCN0000018984 and TRCN0000018987) or control shRNA (SCH002) plasmids were purchased from Sigma and packed as described. Lentiviral particles were precipitated with Lenti-X Concentrator (Clontech) and resuspended in Optimem (Invitrogen). For differentiation protocol MO3.13 cells were seeded at 40% confluence and cultured in regular (DMEM supplemented with 10% FBS) or OL differentiation media (DMEM supplemented with 100 nm phorbol 12-myristate 13-acetate [PMA; Sigma]) for 4 and 10 days respectively with daily media change.

Experiments were carried out 4-5 passages post-transduction. Inhibitors were from: Cayman Chemical Company (A443654, cobimetinib), AdipoGen Life Sciences (selumetinib), Sigma (trametinib). Growth curves were generated using trypan blue exclusion assay and Vi-CellXR (Beckman Coulter).

### Western blot analysis

Whole cell lysates of organs and cells were prepared in 2x SDS lysis buffer (20 mM Tris [pH 7.4], 20 mM EDTA, 2% SDS, 20% glycerol) and concentration determined by DC Protein Assay (Bio-Rad). 30 µg of protein was resolved on 10–20% SDS-PAGE and transferred to PVDF membranes that were blocked and incubated with antibodies diluted in 3% BSA in TBS-T. Binding was detected with enhanced chemiluminescence (Pierce).

### Immunohistochemistry

Organs and tissues were fixed in 4% PFA and embedded in paraffin. Antigen retrieval using heat and citrate buffer was included for all antibodies. Signal was detected with DAB peroxidase substrate (Vector Laboratories). Images were captured on a Nikon Eclipse E600 microscope.

### RNA sequencing

Total RNA was prepared with RNeasy kit (Qiagen) and quality confirmed with Bioanalyzer 2100 (Agilent). TruSeq Stranded Total RNA Library Prep with Ribo-Zero Gold (Illumina, CA, USA) were constructed and sequenced on Illumina HiSeq 2500 at the Hospital for Sick Children (Toronto; paired end 100 bp [E14.5 and human] or 125 bp [human] reads) or Illumina NextSeq 500 at the Princess Margaret Genomics Centre (Toronto; paired end 75 bp reads [mouse tumours]).

Reads were trimmed with Trimmomatic-v0.32^69^ and aligned to GRCm38-v68 (mouse) or GRCh37-v75 (human) using STAR v2.5.0^70^ in two-pass mode; duplicate reads were marked with Picard-v2.5.0. Gene expression was counted with HTSeq^71^, and differential expression calculated with edgeR^72^. Genes with absolute fold-change > 1 and Benjamini-Hochberg adjusted p-value < 0.05 were classed differentially expressed. Pathways were analysed with DAVID^73^. Genes were ranked by multiplying their fold-change sign with the -log10(adjusted p-value) for pre-ranked GSEA^37^, using human homologues of mouse genes obtained from Ensembl. For ssGSEA, reads were aligned to the transcriptome using RSEM-v1.2^74^. Genes with mean FPKM < 1 were discarded, and genes with duplicated names were filtered to keep the most expressed gene. SOX10 target genes were from the Harmonizome^75^. *Fabp7* RNA-Seq expression data was from the Gene Expression Database^76^.

### Network analysis

Transcription factor (TF) networks were scored on three metrics, similar to previous methods^77^, with improvements to network weighting by gene differential expression. Differential gene expression scores were calculated as for pre-ranked GSEA. Networks of TF-DNA and TF-Protein interaction edges were constructed from MARA^78^ and String-DB^79^, respectively, allowing a maximal edge distance of 3 from the root TF. The network score (*N*_*t,n*_) was computed by

$$N_{t,n} = \mathop {\sum }\limits_{r{\it{\epsilon }}V_t} \frac{{S_r \cdot S_n}}{{D_{r,n} \cdot L_{r,n}}}$$

where *r∊V*_*t*_ is the set of all genes (*r*) in the local subnetwork of transcription factor *t*; *S*_*r*_ is the differential expression gene score, *S*_*n*_ is the MARA/String relationship score; *D*_*r,n*_ and *L*_*r,n*_ is the edge distance of gene *r* from *t* and the out-degree of the parent node in the network, respectively. Each TF network was scored as the aggregate rank of each sub-score in decreasing order, such that the higher the rank, the stronger the effect of the TF influence among differentially expressed genes.

### End-point PCR and Q-PCR

Total RNA was reverse transcribed using Reverse Transcription kit (Applied Biosystems). End point PCR was performed for 28 cycles unless indicated otherwise. End-point PCR-validated primers were used for qPCR with iTaq Universal SYBR green supermix (Bio-Rad).

### Mouse exome sequencing

DNA was extracted from frozen tissue samples with DNeasy kit (Qiagen). Exome libraries were generated with the SureSelect Mouse All Exon Kit (Agilent). Paired end 125 bp Illumina HiSeq 2500 sequencing was done at The Hospital for Sick Children. We sequenced a pool of normal CD1 mice because there is no available CD1 reference genome or genome-wide SNP analysis.

Reads were trimmed (Trimmomatic-v0.32^69^) and aligned GRCm38 with bwa-v0.7.8^80^. Indel realignment and base quality recalibration was performed with GATK-v3.6.0^81^ and duplicate reads marked. Sequencing depth was 87-115 × (normal) and 86-134 × (tumour).

Somatic variants were called with VarScan-v2.3.8^82^ and Mutect2^83^, retaining those identified by both. For unmatched tumours, variants were called individually against all normals including the CD1 pool, retaining those identified with both tools against every normal and additionally discarding known variants in other mouse strains, as we could not exclude the possibility that these are also present in a particular CD1 mouse. Variants with >10x coverage in both tumour and normal samples, a minimum tumour variant allele frequency (VAF) of 0.05 and maximum normal VAF of 0.01 were annotated using SnpEFF-v4.3k^84^. Variants were compared with known human cancer variants using the COSMIC-v81^85^. Clonal evolution of tumours with a matched normal was analysed with SuperFreq^86^. Copy number variants were identified using both on- and off-target reads with CNVkit v0.8.6^87^.

### Human exome sequencing

DNA was extracted from frozen tissue samples with DNeasy kit (Qiagen). Libraries were created with Ion TargetSeq Exome 50 Mb library (ThermoFisher), sequenced on Ion Proton machines (ThermoFisher), and aligned to human genome build hg19 with Torrent Suite Software (ThermoFisher) at The Hospital for Sick Children. Variants were called with VarScan and annotated with SnpEFF. RAS pathway and MYC mutant samples were determined by the presence of an alteration in a core RAS/MAPK/PI3K pathway gene (Supplementary Data 4) or *MYC*, respectively. These were restricted to SNVs/indels that introduced missense, frameshift, nonsense or splice site mutations at variant-allele frequency >0.2 and with coverage >20, a copy number gain with 5+ copies, or a homozygous deletion, had to be present in the sample for any pathway gene. Wild-type samples had all pathway genes free of SNV/indels and copy number changes.

### Statistical analysis

Unless otherwise stated, all *p*-values were calculated by two-tailed t-tests, not assuming equal variance between samples.

### Reporting summary

Further information on research design is available in the Nature Research Reporting Summary linked to this article.

## Supplementary information

Supplementary Information

Description of Additional Supplementary Files

Supplementary Data 1

Supplementary Data 2

Supplementary Data 3

Supplementary Data 4

Reporting Summary

## Data Availability

Raw mouse WES and RNA-seq data is available from the Gene Expression Omnibus (GEO), accession GSE120884. Human WES and RNA-seq data is available from the European Genomics Archive, accession EGAD00001006450 (https://ega-archive.org). Publicly available data was from GEO (https://www.ncbi.nlm.nih.gov/geo accessions GSE115875, GSE85390, GSE108364) or https://datahub-jv6f4mbl.udes.genap.ca, and is referenced both within the article and in Supplementary Table S8. All other information supporting the findings of this study are available within the article, its Supplementary Information files, a Source Data file and from the corresponding author upon reasonable request. Source data are provided with this paper.

## Source data

Source Data

## Code availability

The custom python code for performing TF network analysis is available from https://github.com/chawkins-lab/tf_circuit_driver.
