# Supplementary material for: Epigenetic activation of a RAS/MYC axis in H3.3K27M-driven cancer
Source: Nat Commun. 2020 Dec 4;11:6216. doi: 10.1038/s41467-020-19972-7 (PMC7718276; doi:10.1038/s41467-020-19972-7)
Supplement: Supplementary file 1 — Supplementary Information [file 41467_2020_19972_MOESM1_ESM.pdf]

Supplementary Information

Epigenetic activation of a RAS/MYC axis in H3.3K27M-driven cancer

Sanja Pajovic[1,#] Robert Siddaway[1,#] Taylor Bridge[1], Javal Sheth[1], Patricia Rakopoulos[1,2], Byungjin Kim[1,2], Scott Ryall[1,2], Sameer Agnihotri[3], Lauren Phillips[1,2], Man Yu[1], Christopher Li[1,2], Scott Milos[1], Palak Patel[1], Dilakshan Srikanthan[1,2], Annie Huang[1,2,4] and Cynthia Hawkins[1,2,5,*]

1. Arthur and Sonia Labatt Brain Tumour Research Centre, The Hospital for Sick Children, Toronto, Canada
2. Department of Laboratory Medicine and Pathobiology, University of Toronto, Toronto, Canada
3. Department of Neurological Surgery, Children's Hospital of Pittsburgh of UPMC, Pittsburgh, PA 15213, United States
4. Division of Haematology and Oncology, The Hospital for Sick Children, Toronto, Canada
5. Division of Pathology, The Hospital for Sick Children, Toronto, Canada
#. These authors contributed equally to this work
*. Corresponding author: cynthia.hawkins@sickkids.ca

Supplementary Figure 1

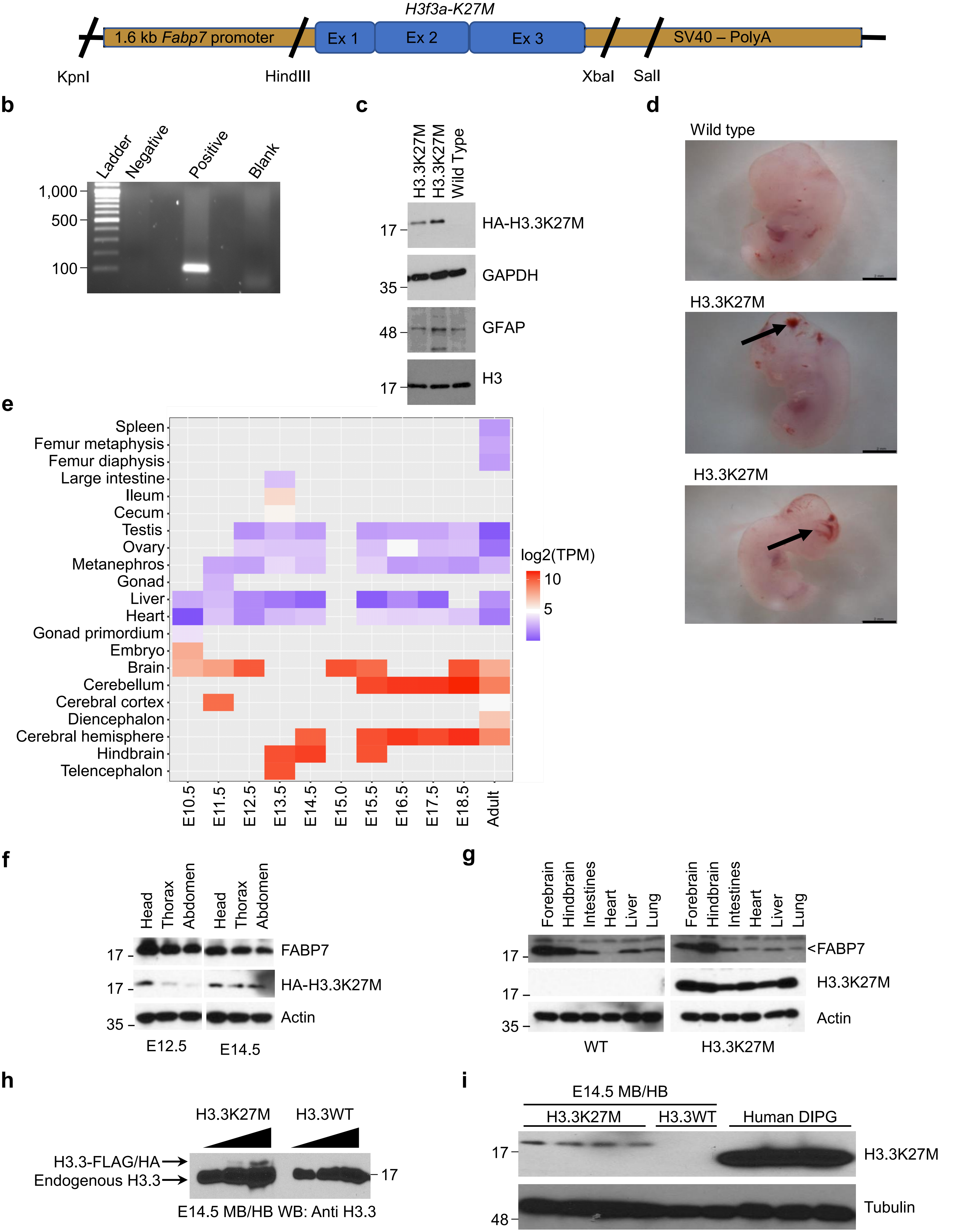

Supplementary Figure 1. Generation and characterization of *Fabp7*-H3.3K27M-FLAG/HA CD1 mice.

A. DNA construct containing histone H3.3K27M cDNA tagged with FLAG/HA at 3' end and directed by the 1.6 kb mouse *Fabp7* promoter. Ex: exon
B. All mice were genotyped with a probe T1 spanning the *Fabp7* promoter and H3.3K27M cDNA. Gels show representative results.
C. Western blot to confirm transgenic H3.3K27M-FLAG/HA expression in E12.5 brains. Results are representative of 2 independent experiments.
D. Photographs of H3.3K27M and WT embryos collected at E12.5 10 pups from 4 litters were examined. Arrows show hemorrhage. Scale bar: 2 mm.
E. Heatmap of *Fabp7* expression in different tissues across various embryonic times and in adult mice. Grid points without a box on the blue-white-red colour scale shown are missing values. Data was retrieved from the Gene Expression Database (http://www.informatics.jax.org/expression).
F. Western blot of head, thorax and abdomen isolated from E12.5 and E14.5 H3.3K27M embryos. Blots representative of 6 independent embryos are shown.
G. Western blot of tissues from H3.3K27M and WT adult mice Results are representative of 4 independent experiments. <: specific band.
H. Western blot of H3.3K27M and WT E14.5 MB/HB (midbrain/hindbrain). Increasing amounts of each sample were loaded. Results are representative of 4 independent experiments.
I. Western blot of H3.3K27M (n=4) and WT E14.5 (n=2) MB/HB and human DIPG (n=3) samples. Blots were repeated 4 times.

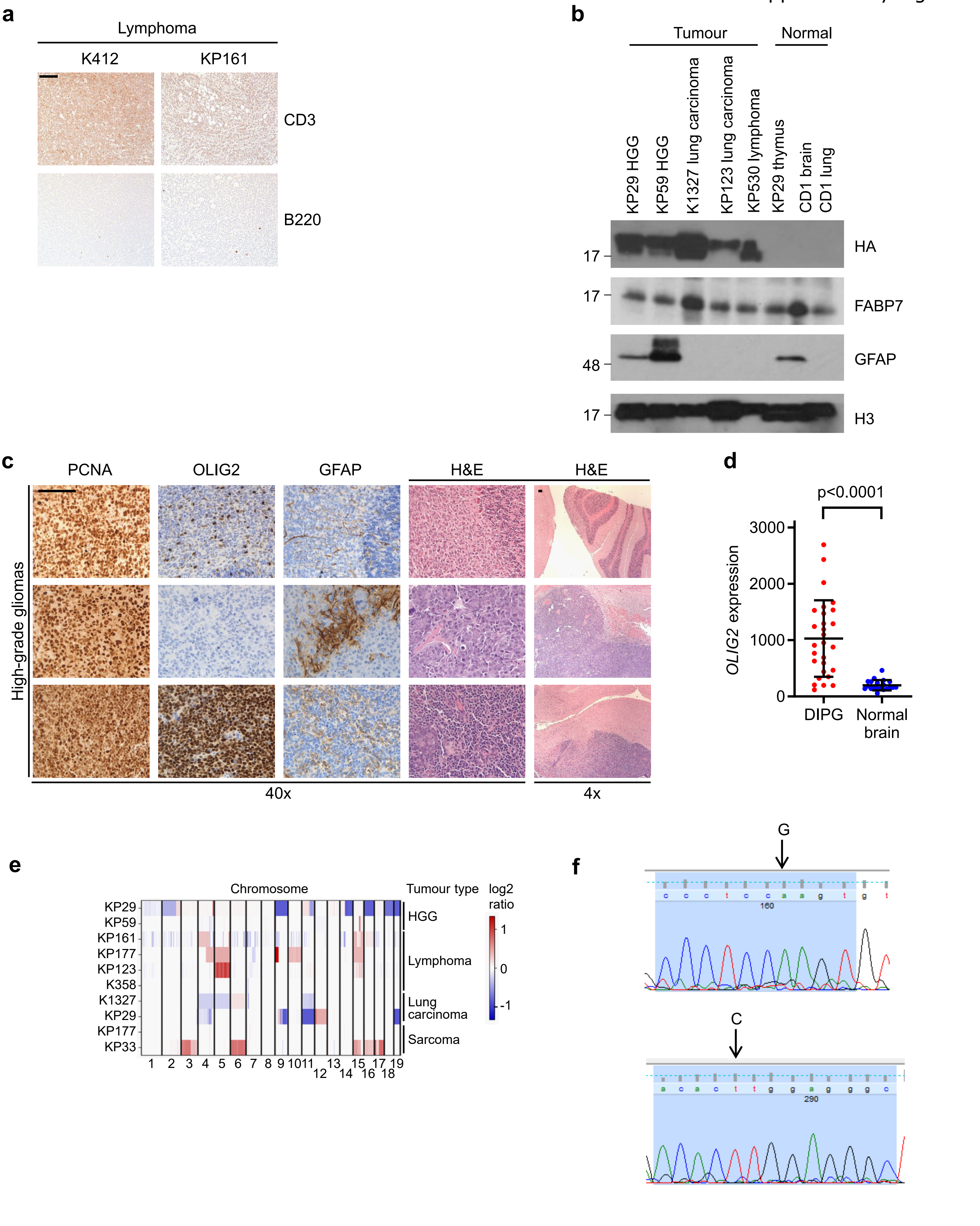
a
Lymphoma
K412
KP161
CD3
B220
b
Tumour
Normal
KP29 HGG
KP59 HGG
K1327 lung carcinoma
KP123 lung carcinoma
KP530 lymphoma
KP29 thymus
CD1 brain
CD1 lung
17
HA
17
FABP7
48
GFAP
17
H3
c
PCNA
OLIG2
GFAP
H&E
H&E
High-grade gliomas
40x
4x
d
p<0.0001
OLIG2 expression
3000
2000
1000
0
DIPG
Normal brain
e
Chromosome
Tumour type
log2 ratio
KP29
KP59
KP161
KP177
KP123
K358
K1327
KP29
KP177
KP33
HGG
Lymphoma
Lung carcinoma
Sarcoma
1
0
-1
1 2 3 4 5 6 7 8 9 10 11 12 13 14 15 16 17 18 19
f
G
C

Supplementary Figure 2. Characterization of H3.3K27M-driven mouse tumours.

A. Immunohistochemistry (IHC) showing H3.3K27M mouse lymphomas are positive for CD3 (T cell lymphoma marker) and negative for B220 (B cell lymphoma marker). Scale bar: 100 μm.
B. Western blot of H3.3K27M mouse tumours and control tissue Blots were repeated at least 2 times.
C. H&E and IHC of H3.3K27M mouse HGGs. Scale bar: 100 μm.
D. *OLIG2* expression in human H3.3K27M DIPG (n=28) and normal brain (n=20). Bars show mean ± standard deviation.
E. Heatmap of log2-fold copy number ratios between normal tissues and H3.3K27M mouse tumours. HGG: high-grade glioma.
F. Sanger sequencing of the *Trp53*-R52Q mutation in K1327 lung carcinoma.

Statistical tests: t (D)

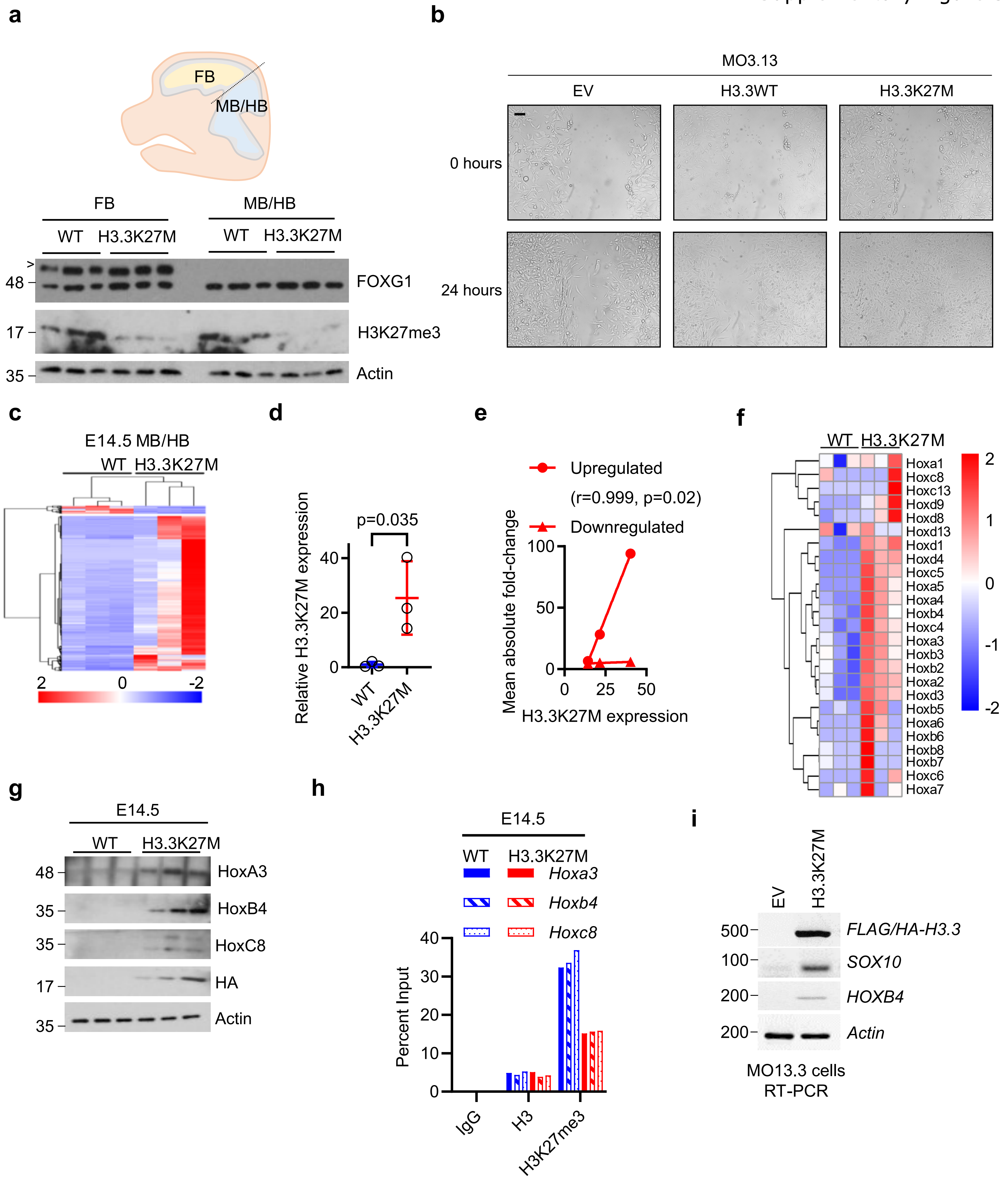
a
FB
MB/HB
FB
MB/HB
WT
H3.3K27M
WT
H3.3K27M
48
17
35
FOXG1
H3K27me3
Actin
b
MO3.13
EV
H3.3WT
H3.3K27M
0 hours
24 hours
c
E14.5 MB/HB
WT
H3.3K27M
2
0
-2
d
p=0.035
Relative H3.3K27M expression
40
20
0
WT
H3.3K27M
e
Upregulated
(r=0.999, p=0.02)
Downregulated
Mean absolute fold-change
100
50
0
0
25
50
H3.3K27M expression
f
WT
H3.3K27M
Hoxa1
Hoxc8
Hoxc13
Hoxd9
Hoxd8
Hoxd13
Hoxd1
Hoxd4
Hoxc5
Hoxa5
Hoxa4
Hoxb4
Hoxc4
Hoxa3
Hoxb3
Hoxb2
Hoxa2
Hoxd3
Hoxb5
Hoxa6
Hoxb6
Hoxb8
Hoxb7
Hoxc6
Hoxa7
2
1
0
-1
-2
g
E14.5
WT
H3.3K27M
48
35
35
17
35
HoxA3
HoxB4
HoxC8
HA
Actin
h
E14.5
WT
H3.3K27M
Hoxa3
Hoxb4
Hoxc8
Percent Input
40
30
20
10
0
IgG
H3
H3K27me3
i
EV
H3.3K27M
500
100
200
200
FLAG/HA-H3.3
SOX10
HOXB4
Actin
MO13.3 cells
RT-PCR

Supplementary Figure 3. Characterization of transcriptome changes in developing MB/HB shortly after H3.3K27M expression.

A. Schematic of brain dissection into mid-brain/hind-brain (MB/HB) and forebrain (FB) regions. Dissection accuracy was assessed by FOXG1 Western blot. Blots show 3 independent embryos. >: specific band.
B. Bright-field images of MO3.13 cells grown to confluence taken immediately and 24 hours after scratching (photographs are representative of 4 independent experiments). Scale bar: 100 μm.
C. Heatmap showing differentially expressed genes (absolute fold-change >2, adjusted p-value <0.05) H3.3K27M E14.5 (n=3) MB/HB compared to WT littermates (n=3).
D. H3.3K27M expression in the E14.5 MB/HB samples used for RNA-Seq was measured by qPCR and normalized to *Gapdh* (n=3).
E. Mean absolute fold-change of up- and down-regulated genes in each H3.3K27M sample was plotted against H3.3K27M expression (n=3).
F. Heatmap of *Hox* gene family expression in WT and H3.3K27M E14.5 MB/HB.
G. Western blot of H3.3K27M and WT E14.5 MB/HB (n=3 independent embryos).
H. ChIP quantifying H3K27me3 in E14.5 WT or H3.3K27M MB/HB. H3 and IgG ChIP are used as controls.
I. RT-PCR analysis of EV and H3.3K27M MO3.13 cells (results are representative of 2 independent experiments).

Statistical tests: t (D,E)

Supplementary Figure 4

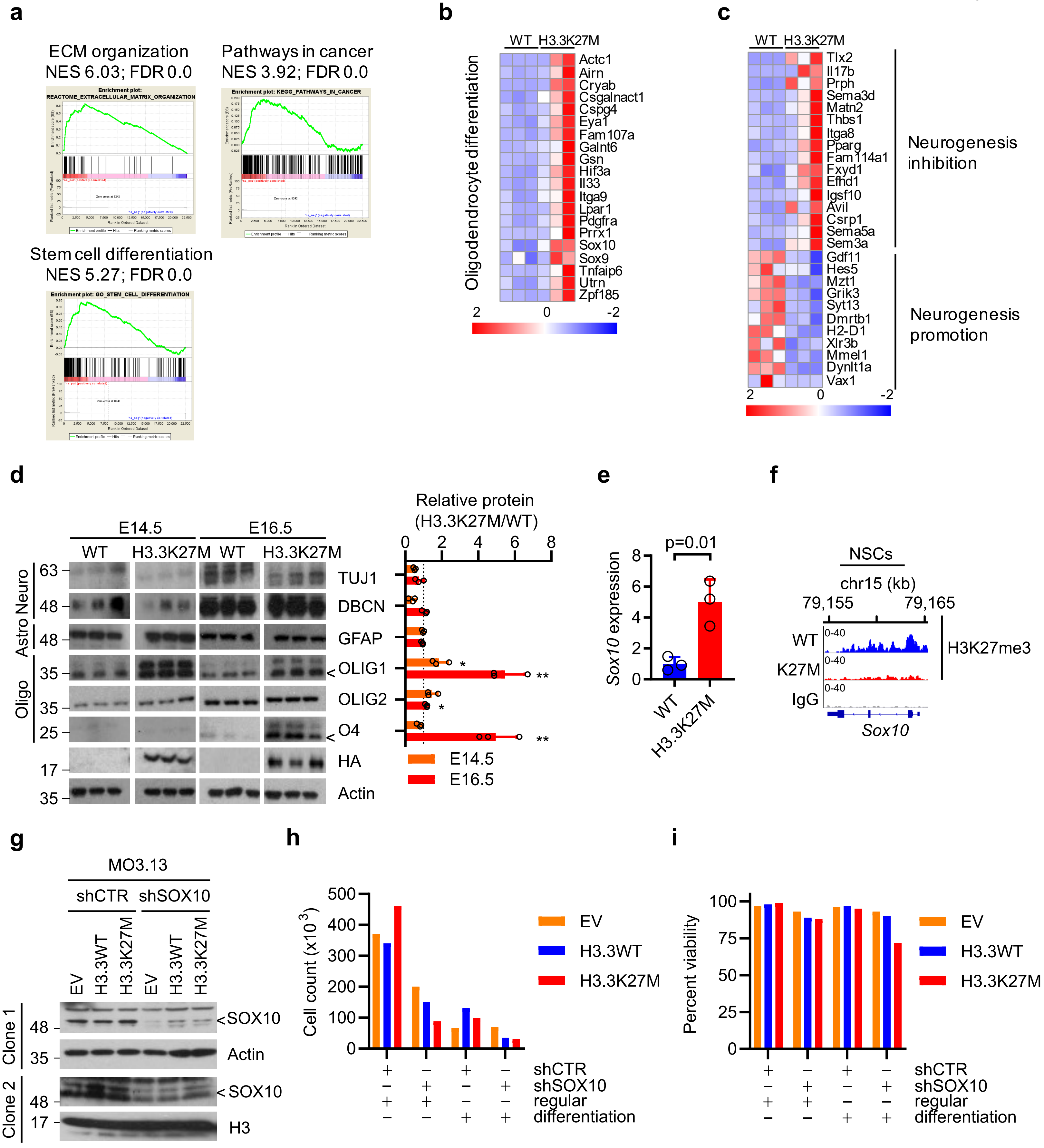

Supplementary Figure 4. Characterization of altered cell-fate specification induced by H3.3K27M.

A. GSEA of upregulated pathways in H3.3K27M E14.5 MB/HB. NES: Normalized Enrichment Score. FDR: false discovery rate. ECM: extracellular matrix.
B. Heatmap of genes involved in OPC/oligodendrocyte commitment that were significantly upregulated (absolute fold-change >2, adjusted p-value <0.05) in H3.3K27M versus WT E14.5 MB/HB.
C. Heatmap of genes involved in neuronal differentiation that were significantly differentially expressed (absolute fold-change >2, adjusted p-value <0.05) in H3.3K27M versus WT E14.5 MB/HB.
D. Western blot of H3.3K27M and WT MB/HB from littermates harvested at E14.5 or E16.5. Densitometry quantifies H3.3K27M versus WT (normalized to actin) and is plotted as mean ± standard deviation (n=3). Oligo: oligodendrocyte, Astro: astrocyte, Neuro: neuronal. <: specific band.
E. *Sox10* expression was measured by qPCR in 3 independent H3.3K27M or WT embryos, normalized to *Gapdh* and WT, and plotted as mean ± standard deviation.
F. H3K27me3 and IgG ChIP-Seq across the *Sox10* promoter and gene body in H3.3WT- or H3.3K27M-expressing NSCs.
G. Western blot of MO3.13 transduced with control shRNA or two independent shSOX10 clones. <: specific band.
H. Cell counts of MO3.13 cells transduced with control (shCTR) or SOX10-specific (shSOX10) shRNA and grown for 4 days in regular or differentiation media
I. Cell counts of MO3.13 cells transduced with control (shCTR) or SOX10-specific (shSOX10) shRNA and grown for 4 days in regular or differentiation media.

Statistical tests: t (D, E) *: p<0.05, **: p<0.01.

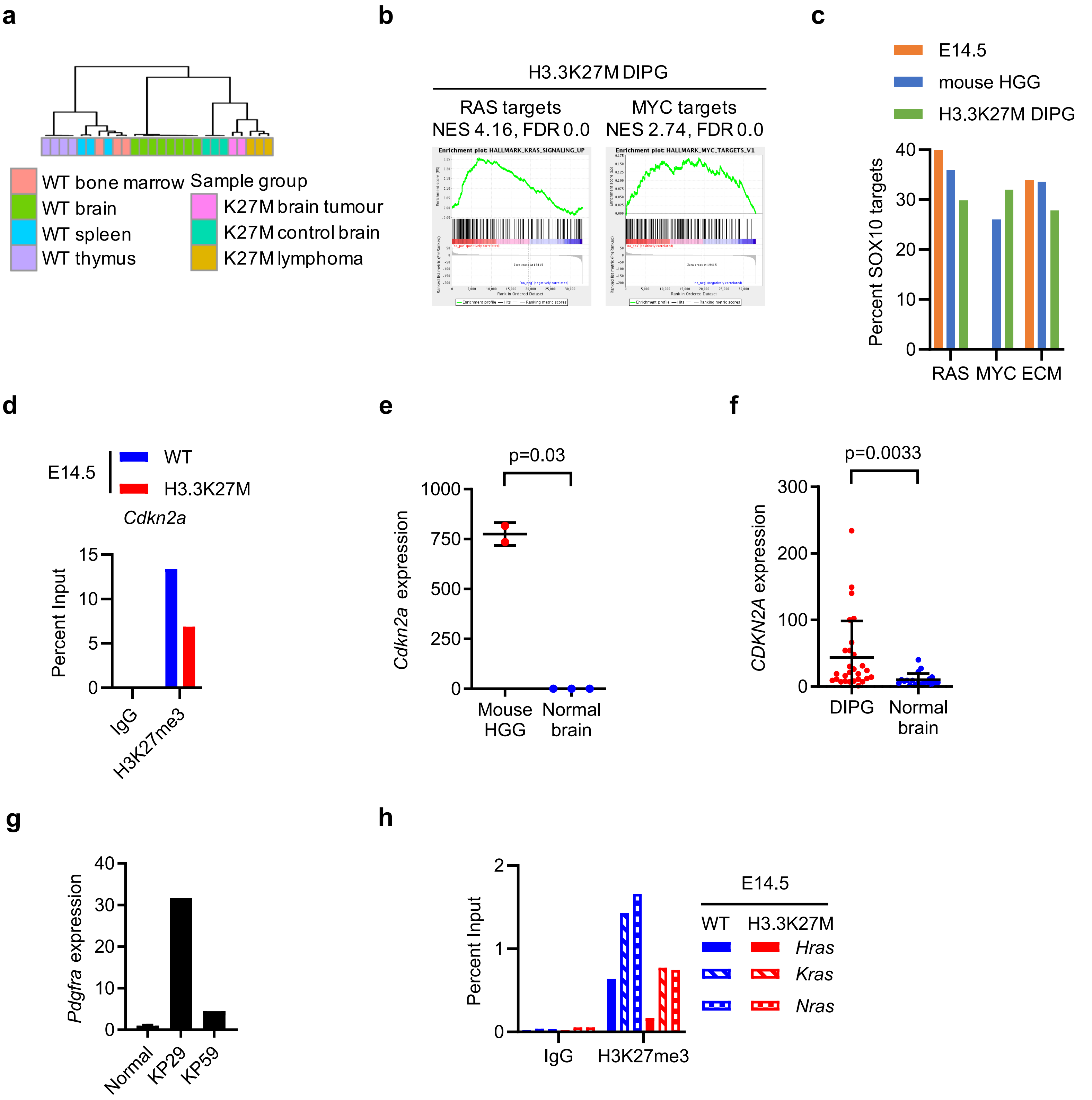
a
WT bone marrow
WT brain
WT spleen
WT thymus
Sample group
K27M brain tumour
K27M control brain
K27M lymphoma
b
H3.3K27M DIPG
RAS targets
NES 4.16, FDR 0.0
MYC targets
NES 2.74, FDR 0.0
Enrichment plot: HALLMARK_KRAS_SIGNALING_UP
Enrichment plot: HALLMARK_MYC_TARGETS_V1
c
E14.5
mouse HGG
H3.3K27M DIPG
Percent SOX10 targets
40
30
20
10
0
RAS
MYC
ECM
d
E14.5
WT
H3.3K27M
Cdkn2a
Percent Input
15
10
5
0
IgG
H3K27me3
e
p=0.03
Cdkn2a expression
1000
750
500
250
0
Mouse HGG
Normal brain
f
p=0.0033
CDKN2A expression
300
200
100
0
DIPG
Normal brain
g
Pdgfra expression
40
30
20
10
0
Normal
KP29
KP59
h
Percent Input
2
1
0
IgG
H3K27me3
E14.5
WT
H3.3K27M
Hras
Kras
Nras

Supplementary Figure 5. Characterization of the H3.3K27M-driven tumour transcriptome.

A. ssGSEA hallmark pathway scores from WT tissues and H3.3K27M healthy brain, HGG and lymphomas were subjected to hierarchical clustering.
B. GSEA of RAS and MYC target genes in H3.3K27M DIPG. NES: Normalized Enrichment Score. FDR: false discovery rate.
C. Overlap of genes differentially expressed at E14.5, in mouse HGG, and in human DIPG that are also SOX10 target genes.
D. ChIP quantifying H3K27me3 at the *Cdkn2a* promoter in E14.5 WT or H3.3K27M MB/HB.
E. *Cdkn2a* expression in mouse H3.3K27M HGG (n=2) and normal brain (n=3). Bars show mean ± standard deviation.
F. *CDKN2A* expression in human H3.3K27M DIPG (n=28) and normal brain (n=20). Bars show mean ± standard deviation.
G. Expression of *Pdgfra* in normal mouse brain (n=3, mean ± standard deviation) plus individual mouse HGG.
H. ChIP quantifying H3K27me3 at the *Hras*, *Kras* and *Nras* promoters in E14.5 WT or H3.3K27M MB/HB.

Statistical tests: t (E,F).

Supplementary Figure 6

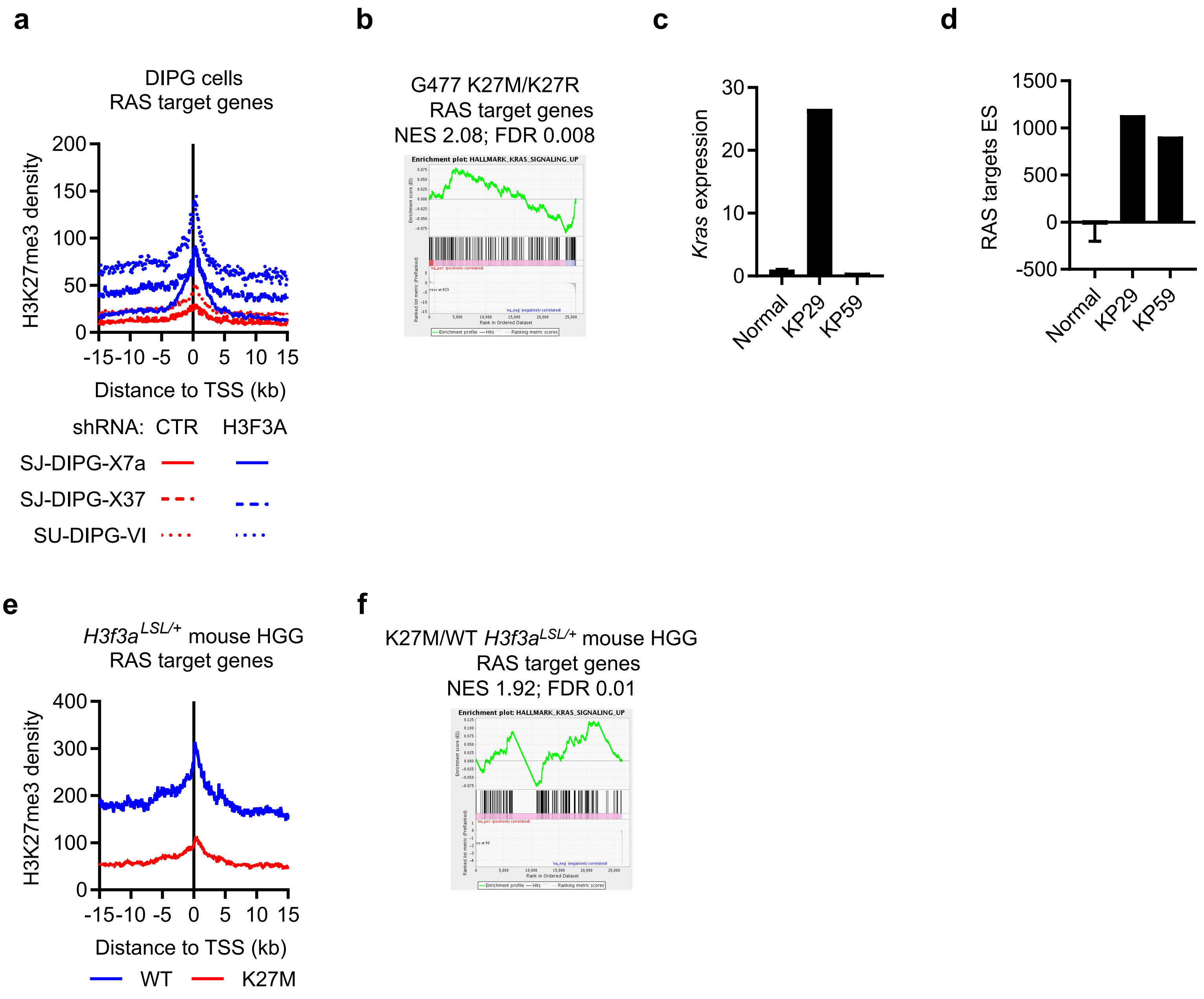

Supplementary Figure 6. Epigenetic activation of RAS/MAPK by H3.3K27M.

A. H3K27me3 ChIP-Seq density was profiled ± 15 kb around the TSS of RAS target genes in DIPG cells transduced with shH3F3A (blue) or control (red) lentivirus. TSS: transcription start site.
B. GSEA of RAS target genes in H3.3K27M versus H3.3K27R G477 cells. NES: Normalized Enrichment Score. FDR: false discovery rate.
C. Expression of *Kras* in normal mouse brain (n=3, mean ± standard deviation) plus individual mouse HGG.
D. ssGSEA of RAS target genes in normal brain (n=3, mean ± standard deviation) plus individual mouse HGG. ES: enrichment score.
E. H3K27me3 ChIP-Seq density was profiled ± 15 kb around the TSS of RAS target genes in HGG derived WT or K27M $H3f3a^{LSL/+}$ mice[16].
F. GSEA of RAS target genes in $H3f3a^{K27M/+}$ versus $H3f3a^{WT/+}$ mice[16].

Supplementary Figure 7

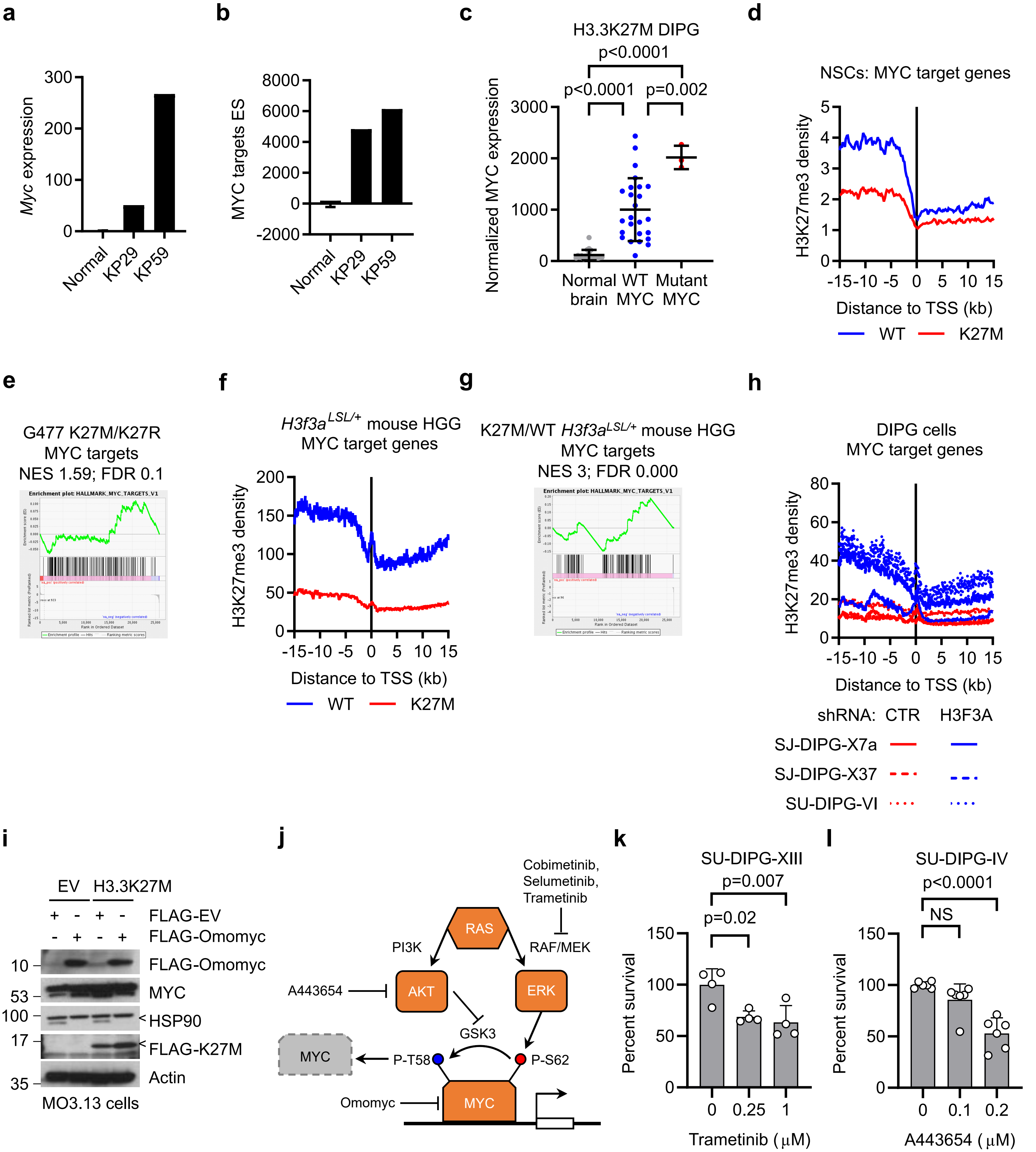

Supplementary Figure 7. Epigenetic activation by H3.3K27M and targeting of MYC.

A. Expression of *Myc* in normal mouse brain (n=3, mean ± standard deviation) plus individual mouse HGG.
B. ssGSEA of MYC target genes in normal brain (n=3, mean ± standard deviation) plus individual mouse HGG. ES: Enrichment score.
C. *MYC* expression in MYC WT (n=25)/mutant (n=3) H3.3K27M DIPG and normal brain. Bars show mean ± standard deviation.
D. H3K27me3 ChIP-Seq density was profiled ± 15 kb around the TSS of MYC target genes in H3.3K27M or H3.3WT NSCs. TSS: transcription start site.
E. GSEA of MYC target genes in H3.3K27M versus H3.3K27R G477 cells. NES: Normalized Enrichment Score. FDR: false discovery rate.
F. H3K27me3 ChIP-Seq density was profiled ± 15 kb around the TSS of RAS target genes in HGG derived WT or K27M $H3f3a^{LSL/+}$ mice.
G. GSEA of RAS target genes in $H3f3a^{K27M/+}$ versus $H3f3a^{WT/+}$ mice.
H. H3K27me3 ChIP-Seq density was profiled ± 15 kb around the TSS of RAS target genes in DIPG cells transduced with shH3F3A (blue) or control (red) lentivirus.
I. Western blot of MO3.13 cells 3 days post-transfection with FLAG-EV or FLAG-Omomyc plasmids (results are representative of 3 independent experiments). <: specific band.
J. Schematic of RAS/MYC axis and treatments used in this study.
K. Viability assay of primary SU-DIPG-XIII cells treated or not with trametinib for 3 days. Bars show mean ± standard deviation (n=4).
L. Viability assay of primary SU-DIPG-IV cells treated or not with A443654 for 3 days. Bars show mean ± standard deviation (n=6).

Statistical tests: ANOVA (C), t (K, L).

Supplementary Figure 8

a

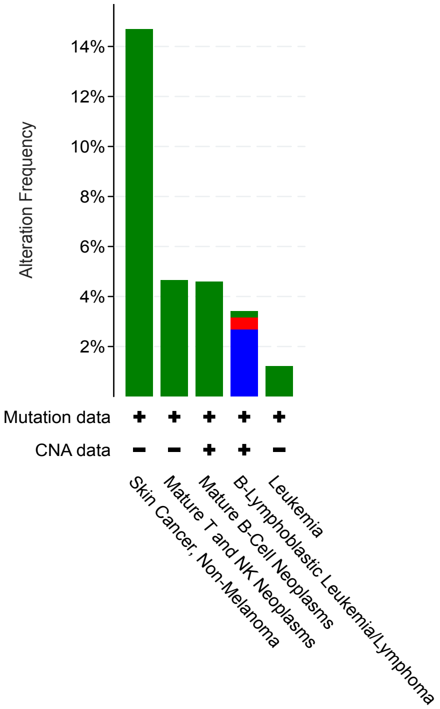

b

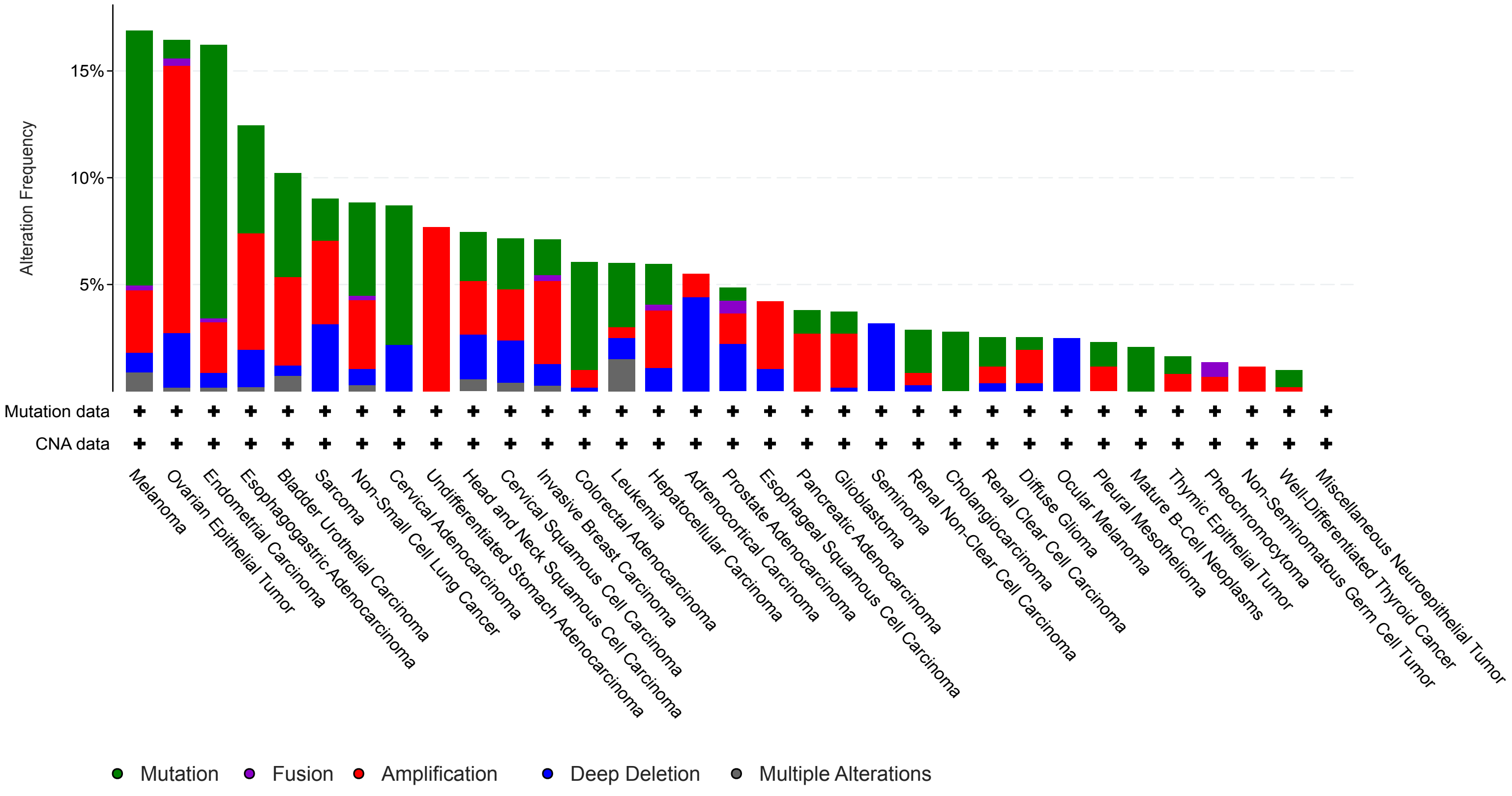

Supplementary Figure 8. PRC2 mutations in human cancers

A. Core PRC2 (EZH1, EZH2, EED, SUZ12) mutations in the indicated tumour types, summarised by combining non-redundant datasets from cBioPortal.
B. Core PRC2 (EZH1, EZH2, EED, SUZ12) mutations in the indicated tumour types using non-redundant datasets with a minimum of 50 samples from cBioPortal.

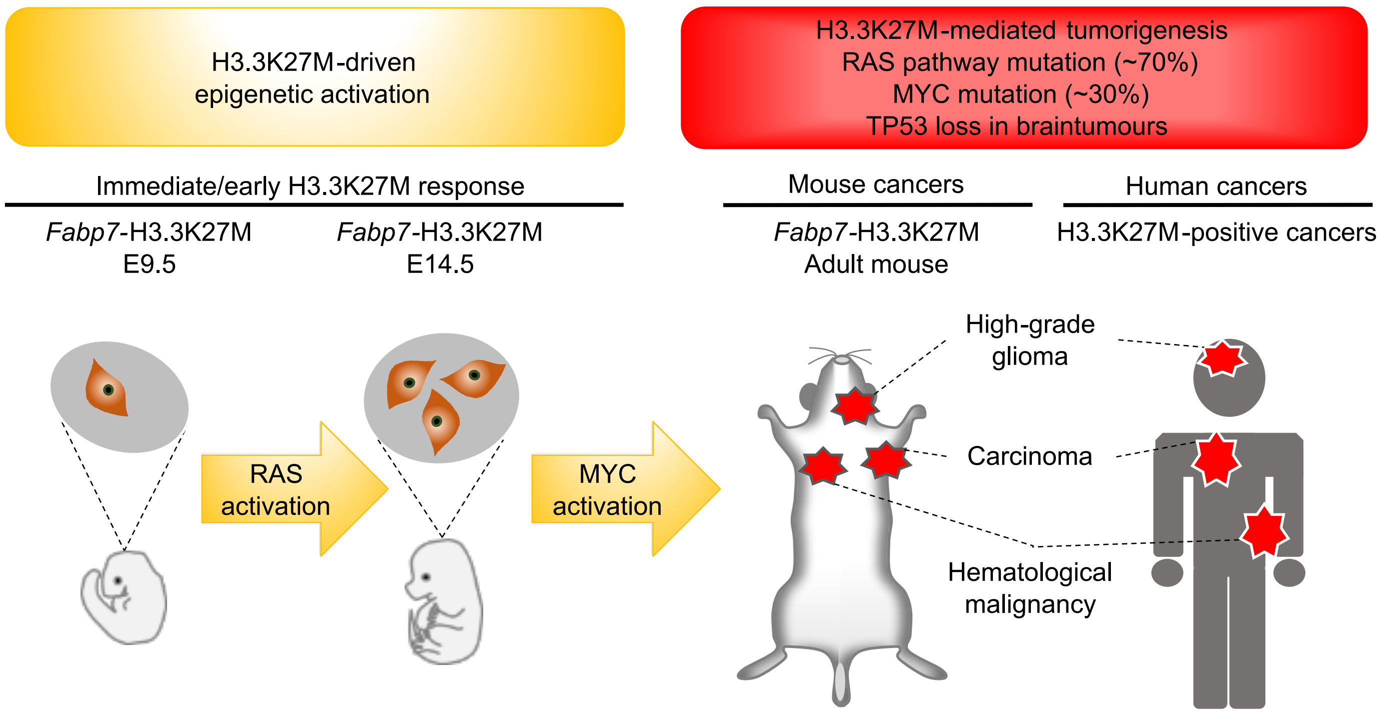
H3.3K27M-driven
epigenetic activation
H3.3K27M-mediated tumorigenesis
RAS pathway mutation (~70%)
MYC mutation (~30%)
TP53 loss in braintumours
Immediate/early H3.3K27M response
Mouse cancers
Human cancers
Fabp7-H3.3K27M
E9.5
Fabp7-H3.3K27M
E14.5
Fabp7-H3.3K27M
Adult mouse
H3.3K27M-positive cancers
RAS
activation
MYC
activation
High-grade
glioma
Carcinoma
Hematological
malignancy

Supplementary Figure 9. Model of epigenetic activation of a RAS/MYC axis in H3.3K27M-driven cancers.

Supplementary Tables

Table S1: RNA-Seq differential expression for mouse E14.5 and brain tumors, and commonly regulated pathways
Table S2: Survival and tumour characteristics of mice in the study
Table S3: DIPG patient characteristics
Table S4: Core RAS/MAPK/PI3K pathway genes
Tables S1-4 are provided as supplementary spreadsheets.
Table S5: Antibodies

Antibody	Vendor/cat#	Dilution
Actin	Sigma A2066	1:5,000
AKT	Cell Signaling #4691	1:1,000
Phospho-AKT (S473)	Cell Signaling #9271	1:1,000
Cleaved Caspase-3	Cell Signaling #9661	1:1,000
Cleaved Caspase-3	R&D systems MAB835	1:1,000
Cleaved Caspase-8	Novus Biologicals NB100-56116SS	1:1,000
Cleaved Caspase-12	Novus Biologicals NBP1-76801SS	1:1,000
DBCN (Doublecortin)	Novus Biologicals NBP1-92684	1:5,000
ERK1/2	Cell Signaling #9102	1:3,000
Phospho-ERK1/2 (T202, Y204)	Cell Signaling #9101	1:1,000
FABP7	Novus Biologicals NBP2-16399	1:1,000
FABP7	Abcam ab32423	1:1,000
FLAG tag	Sigma F7425	1:5,000
FOXG1	Abcam ab18259	1:3,000
GAPDH	Cell Signaling #5174	1:10,000
GFAP	DAKO Z0334	1:5,000
HA tag	Rockland 600-401-384	1:3,000
HA tag	Covance MMS-101P	1:5,000
HA tag	Millipore 05-904	1:5,000
Phospho-H3(Ser10)	Millipore 06-570	1:5,000
Trimethyl-H3(Lys27)	Millipore 07-449	1:5,000
H3	Cell Signaling #9715	1:5,000
H3.3	Abcam ab176840	1:5,000
H3K27M	Millipore ABE419	1:1,000
H3K27M	Abcam ab190631	1:5,000
HOXA3	Santa Cruz Biotechnology sc-374237	1:1,000
HOXB4	Abcam ab133521	1:1,000
HOXC8	Santa Cruz Biotechnology sc-517007	1:1,000
HSP90	Santa Cruz Biotechnology sc-13119	1:10,000
Lymphotoxin beta	Novus Biologicals AF1008	1:500
MEK1/2	Abcam ab178876	1:10,000
Phospho-MEK1/2 (S217, S221)	Cell signalling 2338S	1:3,000
S100-β	Novus Biologicals NBP-45267	1:1,000

MBP	Novus Biologicals NBP-22121	1:1,0000
MYC	Novus Biologicals NBP2-43627	1:1,000
Phospho-MYC (T58)	Abcam ab28842	1:1,000
Phospho-MYC (S62)	Cell signalling 13748S	1:1,000
OLIG1	Novus Biologicals MAB2417-SP	1:1,000
OLIG2	Sigma Life Science HPA003254	1:1,000
Oligodendrocyte O4 marker	Novus Biologicals MAB1326	1:2,000
PCNA	DAKO M0879	1:10,000
PDGFRA	Millipore 07-276	1:3,000
PARP	Abcam ab6079-1	1:3,000
SOX10	Novus Biologicals NBP2-59620	1:1,000
TUJ1	Novus Biologicals NBP1-42568SS	1:5,000

Table S6: Q-PCR primers

Primer	Species	Sequence
Transgene full F	Mouse	ATG GCT CGT ACA AAG CAG ACT GCC
Transgene full R	Mouse	CTAGGCGTAGTCGGGCACGTCGTA
Transgene short F	Mouse	ACTGAACTTCTGATCCGCAAGCTC
Transgene short R	Mouse	CTTGTCATCGTCGTCCTTGTAGTC
Gfap F	Mouse	CGTGTGGATTTGGAGAGAAAG
Gfap R	Mouse	GTGAGGTCTGCAAACTTAGACC
Pdgfra F	Mouse	GGAGACTCAAGTAACCTTGCAC
Pdgfra R	Mouse	TCAGTTCTGACGTTGCTTTCAA
Sox10 F	Mouse	ACAATGCTGAGCTCAGCAAGACAC
Sox10 R	Mouse	TACTTGTAGTCCGGATGGTCCTT
Fabp7 F	Mouse	AAGTGGGAAACGTGACCAAAC
Fabp7 R	Mouse	CAACCGAACCACAGACTTACAG
Actb F	Mouse	CGCCACCAGTTCGCCATGGA
Actb R	Mouse	TACAGCCCGGGGAGCATCGT
Gapdh F	Mouse	AAGGTCATCCCAGAGCTGAA
Gapdh R	Mouse	CTGCTTCACCACCTTCTTGA
MOBP F	Human	GTGTTCGGAGTACTTCTGGTT
MOBP R	Human	GCAAATGATACCAAGACAAGCTC
GALC F	Human	TGTCGTGACCTGGATTGTGG
GALC R	Human	TGACCTCTCATTCCAAATTCCA
PLP1 F	Human	GGTTTCCCTGCTCACCTTCA
PLP1 R	Human	TCAGAACTTGGTGCCTCGG
CNP F	Human	ATGGTCAGCGTGAAGGC
CNP R	Human	CAACCAAGTTTTGTGACTACGG
S100b F	Human	ATTCTGGAAGGGAGGGAGAC
S100b R	Human	CGTGGCAGGCAGTAGTAACC
GAPDH F	Human	CGACCACTTTGTCAAGCTCA

GAPDH R	Human	AGGGGTCTACATGGCAACTG
ACTB F	Human	GTCTTCCCCTCCATCGTG
ACTB R	Human	AGGGTGAGGATGCCTCTCTT

Table S7: Mouse ChIP Q-PCR primers

Target	Forward	Reverse
Sox10	CCCACACCTAGAGACGGTTG	CTGCGCTAAACCCACTCG
Pdgfra	GCACCAGGAGAAGTGTGTGT	CCCTTCACACCTCCCTTTTT
Hoxa3	AATTACCTCCCTGCATCTCAAA	TTATCAGAGCAGACCCACAATG
Hoxb4	ATTTCCTTATCCGGGAATCG	GTTTCCGAAAGCCCTCCTAC
Hoxc8		
Hras	GATTGGTCGAACTCCTCCGC	GCTTCGGCTTCTGACGGATG
Nras	CACTAGCACCTAGCGCTTTCA	CGACGACTAGAGTCTTGCAGC
Kras	GCTGGCGCTCCCGATAG	GAGGAGCAGCAGCGGG
Cdkn2a	CTGTTTCAACGCCCAGCTCTC	CTGTTTCAACGCCCAGCTCTC

Table S8: Cell lines

Cell line	Mutations	ChIP-Seq normalization	NGS PMID
BT245	H3K27M, *TP53*, *TERT* promoter, *MYC* amp	Drosophila spike-in	30890717
SU-DIPG-XIII	H3K27M, *TP53*, *MYCN* amp	Drosophila spike-in	30890717
G477	H3WT	Drosophila spike-in	30890717
HSJ019	H3WT	Drosophila spike-in	30890717
pcGBM2	H3WT, *TP53*, *EGFR* amp	Drosophila spike-in	30890717
SJ-DIPG-X7a	H3K27M	Mouse cells trapped in xenograft/Drosophila spike-in	30770999
SJ-DIPG-X37	H3K27M	Mouse cells trapped in xenograft/Drosophila spike-in	30770999
SU-DIPG-VI	H3K27M, *TP53*	Mouse cells trapped in xenograft/Drosophila spike-in	30770999
SU-DIPG-IV	H3K27M, *ACVR1*	N/A	N/A
SU-DIPG-XVII	H3K27M	N/A	N/A
SU-DIPG-XXXVI		N/A	N/A
SF8628	H3K27M	N/A	N/A
NSC (mouse)	H3K27M+PDGFB	Background read densities	28263309
