# Supplementary material for: Epigenetic activation of a RAS/MYC axis in H3.3K27M-driven cancer
Source: Nat Commun. 2020 Dec 4;11:6216. doi: 10.1038/s41467-020-19972-7 (PMC7718276; doi:10.1038/s41467-020-19972-7)
Supplement: Supplementary file 2 — Description of Additional Supplementary Files [file 41467_2020_19972_MOESM2_ESM.pdf]

Description of Additional Supplementary Files

Title: Supplementary Data 1

Description: Table S1, RNA-Seq differential expression for mouse E14.5 and brain tumors, and commonly regulated pathways

Title: Supplementary Data 2

Description: Table S2, Survival and tumour characteristics of mice in the study

Title: Supplementary Data 3

Description: Table S3, DIPG patient characteristics

Title: Supplementary Data 4

Description: Table S4, Core RAS/MAPK/PI3K pathway genes
