# Supplementary material for: Epigenetic activation of a RAS/MYC axis in H3.3K27M-driven cancer
Source: Nat Commun. 2020 Dec 4;11:6216. doi: 10.1038/s41467-020-19972-7 (PMC7718276; doi:10.1038/s41467-020-19972-7)
Supplement: Supplementary file 7 — Reporting Summary [file 41467_2020_19972_MOESM7_ESM.pdf]

nature research

Corresponding author(s): Cynthia Hawkins

Last updated by author(s): Nov 2, 2020

Reporting Summary

Nature Research wishes to improve the reproducibility of the work that we publish. This form provides structure for consistency and transparency in reporting. For further information on Nature Research policies, see our Editorial Policies and the Editorial Policy Checklist.

Statistics

For all statistical analyses, confirm that the following items are present in the figure legend, table legend, main text, or Methods section.

n/a	Confirmed	
☐	☒	The exact sample size (*n*) for each experimental group/condition, given as a discrete number and unit of measurement
☐	☒	A statement on whether measurements were taken from distinct samples or whether the same sample was measured repeatedly
☐	☒	The statistical test(s) used AND whether they are one- or two-sided<br>*Only common tests should be described solely by name; describe more complex techniques in the Methods section.*
☒	☐	A description of all covariates tested
☐	☒	A description of any assumptions or corrections, such as tests of normality and adjustment for multiple comparisons
☐	☒	A full description of the statistical parameters including central tendency (e.g. means) or other basic estimates (e.g. regression coefficient) AND variation (e.g. standard deviation) or associated estimates of uncertainty (e.g. confidence intervals)
☐	☒	For null hypothesis testing, the test statistic (e.g. *F*, *t*, *r*) with confidence intervals, effect sizes, degrees of freedom and *P* value noted<br>*Give P values as exact values whenever suitable.*
☒	☐	For Bayesian analysis, information on the choice of priors and Markov chain Monte Carlo settings
☒	☐	For hierarchical and complex designs, identification of the appropriate level for tests and full reporting of outcomes
☒	☐	Estimates of effect sizes (e.g. Cohen's *d*, Pearson's *r*), indicating how they were calculated

Our web collection on statistics for biologists contains articles on many of the points above.

Software and code

Policy information about availability of computer code

Data collection	No software was used.
Data analysis	Trimmomatic-v0.32, STAR v2.5.0, Picard-v2.5.0, edgeR, DESeq, Rtsne-v0.15, DAVID v6.8, GSEA v3.0, ssSSEA (GenePattern), RSEM-v1.2, ISMARA (https://ismara.unibas.ch/mara/), bwa-v0.7.8, GATK-v3.6.0, VarScan-v2.3.8, Mutect2, SnpEFF-v4.3k, SuperFreq, CNVkit v0.8.6, R-v3.5, GraphPad Prism 8, tf_circuit_driver (https://github.com/chawkins-lab/tf_circuit_driver), bedtools-v2.27.1, python-v3

For manuscripts utilizing custom algorithms or software that are central to the research but not yet described in published literature, software must be made available to editors and reviewers. We strongly encourage code deposition in a community repository (e.g. GitHub). See the Nature Research guidelines for submitting code & software for further information.

Data

Policy information about availability of data

All manuscripts must include a data availability statement. This statement should provide the following information, where applicable:

- Accession codes, unique identifiers, or web links for publicly available datasets
- A list of figures that have associated raw data
- A description of any restrictions on data availability

Raw mouse WES and RNA-seq data is available from the Gene Expression Omnibus (GEO), accession GSE120884 (https://www.ncbi.nlm.nih.gov/geo/query/acc.cgi?acc=GSE120884). Human WES and RNA-seq data is available from the European Genomics Archive, accession EGAD00001006450 (https://ega-archive.org). Publicly available data was from GEO (https://www.ncbi.nlm.nih.gov/geo accessions GSE115875, GSE85390, GSE108364) or https://datahub-jv6f4mbl.udes.genap.ca, and is referenced both within the article and in Table S8. All other information supporting the findings of this study are available within the article, its supplementary information files, a Source Data file and from the corresponding author upon reasonable request.
Code for performing TF network analysis is available from https://github.com/chawkins-lab/tf_circuit_driver.

Field-specific reporting

Please select the one below that is the best fit for your research. If you are not sure, read the appropriate sections before making your selection.

☒ Life sciences ☐ Behavioural & social sciences ☐ Ecological, evolutionary & environmental sciences

For a reference copy of the document with all sections, see nature.com/documents/nr-reporting-summary-flat.pdf

Life sciences study design

All studies must disclose on these points even when the disclosure is negative.

Sample size	Sample sizes for animals were determined according to previous studies performed by our group and other scholars with a minimum requirement of 5 mice per genotype. The number of samples reported on in each section is stated in either text or figure legend and varied by genotype depending on live births from breeding. By genotype, we analysed H3.3K27M (n=36), Trp53-/- (n=7 plus n=19 publicly available), H3.3K27M/Trp53+/- (n=24), H3.3K27M/Trp53-/- (n=25) and CD1 (publicly available, n=662) mice.
Data exclusions	No data were excluded from analyses.
Replication	Mouse colonies were derived from three independent founders. Similar results were observed with all three. In vitro experiments were performed in triplicate unless otherwise stated. Replication information is included in the Figure Legends and/or text. All replication attempts were successful.
Randomization	Not applicable: mice were not separated into different arms during the study.
Blinding	Blinding of researchers was not carried out: no mice were treated during the study. Humane endpoint monitoring of all mice of all genotypes was carried out by the same member of staff at the animal facility according to pre-defined and approved methods (as defined by The Hospital for Sick Children Animal Care Committee). This ensured consistent treatment of all genotypes.

Reporting for specific materials, systems and methods

We require information from authors about some types of materials, experimental systems and methods used in many studies. Here, indicate whether each material, system or method listed is relevant to your study. If you are not sure if a list item applies to your research, read the appropriate section before selecting a response.

Materials & experimental systems

n/a	Involved in the study	
☐	☒	Antibodies
☐	☒	Eukaryotic cell lines
☒	☐	Palaeontology and archaeology
☐	☒	Animals and other organisms
☐	☒	Human research participants
☒	☐	Clinical data
☒	☐	Dual use research of concern

Methods

n/a	Involved in the study	
☒	☐	ChIP-seq
☒	☐	Flow cytometry
☒	☐	MRI-based neuroimaging

Antibodies

Antibodies used

1 Actin Sigma A2066
2 AKT Cell Signaling 4691
3 Phospho-AKT (S473) Cell Signaling 9271
4 DBCN (Doublecortin) Novus Biologicals NBP1-92684
5 ERK1/2 Cell Signaling 9102
6 Phospho-ERK1/2 (T202, Y204) Cell Signaling 9101
7 FABP7 Novus Biologicals NBP2-16399
8 FABP7 Abcam ab32423
9 FLAG tag Sigma F7425
10 FOXG1 Abcam ab18259
11 GAPDH Cell Signaling 5174
12 GFAP DAKO Z0334
13 HA tag Rockland 600-401-384
14 HA tag Covance MMS-101P
15 HA tag Millipore 05-904
16 Phospho-H3(Ser10) Millipore 06-570
17 Trimethyl-H3(Lys27) Millipore 07-449

18 H3 Cell Signaling 9715
19 H3K27M Millipore ABE419
20 H3K27M Abcam ab190631
21 HOXA3 Santa Cruz Biotechnology sc-374237
22 HOXB4 Abcam ab133521
23 HOXC8 Santa Cruz Biotechnology sc-517007
24 HSP90 Santa Cruz Biotechnology sc-13119
25 Lymphotoxin beta Novus Biologicals AF1008
26 MEK1/2 Abcam ab178876
27 Phospho-MEK1/2 (S217, S221) Cell signalling 2338
28 S100-β Novus Biologicals NBP-45267
29 MBP Novus Biologicals NBP-22121
30 MYC Novus Biologicals NBP2-43627
31 Phospho-MYC (T58) Abcam ab28842
32 Phospho-MYC (S62) Cell signalling 13748
33 OLIG1 Novus Biologicals MAB2417
34 OLIG2 Sigma HPA003254
35 Oligodendrocyte O4 marker Novus Biologicals MAB1326
36 PCNA DAKO M0879
37 PDGFRA Millipore 07-276
38 SOX10 Novus Biologicals NBP2-59620
39 TUJ1 Novus Biologicals NBP1-42568
40 H3.3 Abcam ab176840

Validation

1 Actin https://www.sigmaaldrich.com/catalog/product/sigma/a2066
2 AKT https://www.cellsignal.com/products/primary-antibodies/akt-pan-c67e7-rabbit-mab/4691
3 Phospho-AKT (S473) https://www.cellsignal.com/products/primary-antibodies/phospho-akt-ser473-antibody/9271
4 DBCN (Doublecortin) https://www.novusbio.com/products/doublecortin-antibody-3e1_nbp1-92684
5 ERK1/2 https://www.cellsignal.com/products/primary-antibodies/p44-42-mapk-erk1-2-antibody/9102
6 Phospho-ERK1/2 (T202, Y204) https://www.cellsignal.com/products/primary-antibodies/phospho-p44-42-mapk-erk1-2-thr202-tyr204-antibody/9101
7 FABP7 https://www.novusbio.com/products/fabp7-b-fabp-antibody_nbp2-16399
8 FABP7 https://www.abcam.com/blbp-antibody-ab32423.html
9 FLAG tag https://www.sigmaaldrich.com/catalog/product/sigma/f7425?lang=en&region=US
10 FOXG1 https://www.abcam.com/foxg1-antibody-ab18259.html
11 GAPDH https://www.cellsignal.com/products/primary-antibodies/gapdh-d16h11-xp-rabbit-mab/5174
12 GFAP https://www.chem.agilent.com/store/productDetail.jsp?catalogId=Z033429-2
13 HA tag https://rockland-inc.com/store/Antibodies-to-More-Epitope-Tags-600-401-384-O4L_12406.aspx
14 HA tag https://www.biolegend.com/en-us/products/purified-anti-ha-11-epitope-tag-antibody-11374
15 HA tag https://www.emdmillipore.com/CA/en/product/Anti-HA-Tag-Antibody,MM_NF-05-904
16 Phospho-H3(Ser10) https://www.emdmillipore.com/CA/en/product/Anti-phospho-Histone-H3-Ser10-Antibody-Mitosis-Marker,MM_NF-06-570
17 Trimethyl-H3(Lys27) https://www.emdmillipore.com/CA/en/product/Anti-trimethyl-Histone-H3-Lys27-Antibody,MM_NF-07-449
18 H3 https://www.cellsignal.com/products/primary-antibodies/histone-h3-antibody/9715?Ntk=Products&Ntt=9715
19 H3K27M https://www.sigmaaldrich.com/catalog/product/mm/abe419
20 H3K27M https://www.abcam.com/histone-h3-mutated-k27m-antibody-epr18340-chip-grade-ab190631.html
21 HOXA3 https://www.scbt.com/p/hoxa3-antibody-f-7
22 HOXB4 https://www.abcam.com/hoxb4-antibody-epr1917-ab133521.html
23 HOXC8 https://www.scbt.com/p/hoxc8-antibody-1h2
24 HSP90 https://www.scbt.com/p/hsp-90alpha-beta-antibody-f-8
25 Lymphotoxin beta https://www.novusbio.com/products/lymphotoxin-beta-r-tnfrsf3-antibody_af1008
26 MEK1/2 https://www.abcam.com/mek1--mek2-antibody-epr16667-ab178876.html
27 Phospho-MEK1/2 (S217, S221) https://www.cellsignal.com/products/primary-antibodies/phospho-mek1-2-ser221-166f8-rabbit-mab/2338
28 S100-β https://www.novusbio.com/products/s100b-antibody-15f9nb_nbp2-45267
29 MBP https://www.novusbio.com/products/mbp-antibody-2h9_nbp2-22121
30 MYC https://www.novusbio.com/products/myc-epitope-tag-antibody-2_nbp2-43627
31 Phospho-MYC (T58) https://www.abcam.com/c-myc-phospho-t58-antibody-ab28842.html
32 Phospho-MYC (S62) https://www.cellsignal.com/products/primary-antibodies/phospho-c-myc-ser62-e1j4k-rabbit-mab/13748
33 OLIG1 https://www.novusbio.com/products/olig1-antibody-257219_mab2417
34 OLIG2 https://www.sigmaaldrich.com/catalog/product/sigma/hpa003254
35 Oligodendrocyte O4 marker https://www.novusbio.com/products/oligodendrocyte-marker-o4-antibody-o4_mab1326
36 PCNA https://www.agilent.com/en/product/immunohistochemistry/antibodies-controls/primary-antibodies/proliferating-cell-nuclear-antigen-(concentrate)-76551
37 PDGFRA https://www.emdmillipore.com/US/en/product/Anti-PDGFR-Antibody,MM_NF-07-276
38 SOX10 https://www.novusbio.com/products/sox10-antibody-sox10-991_nbp2-59620
39 TUJ1 https://www.novusbio.com/products/beta-iii-tubulin-antibody-2e9_nbp1-42568

	40 H3.3 https://www.abcam.com/histone-h33-antibody-epr17899-chip-grade-ab176840.html

Eukaryotic cell lines

Policy information about cell lines

Cell line source(s)	Cedarlane: MO3.13<br>ATCC: HEK293T<br>Millipore: SF8628<br>Michele Monje (Stanford University): SU-DIPG-IV, SU-DIPG-VI, SU-DIPG-XIII, SU-DIPG-XVII and SU-DIPG-XXXVI
Authentication	Cell lines were authenticated by the supplier.
Mycoplasma contamination	All cell lines were routinely tested for mycoplasma. No contaminations were detected.
Commonly misidentified lines (See ICLAC register)	HEKT293T cells were used for lentiviral production.

Animals and other organisms

Policy information about studies involving animals; ARRIVE guidelines recommended for reporting animal research

Laboratory animals	Mouse strains used were: ICR-CD1, B6.129S2-Trp53tm1Tyj/J. Both female and male mice were utilised from embryonic timepoints to endpoint (this was either a humane endpoint or at a time to harvest embryos at a specific developmental time point).
Wild animals	The study did not involve wild animals
Field-collected samples	The study did not involve field-collected samples
Ethics oversight	The Hospital for Sick Children Animal Care Committee

Note that full information on the approval of the study protocol must also be provided in the manuscript.

Human research participants

Policy information about studies involving human research participants

Population characteristics	Samples were taken from fully-consented patients diagnosed with DIPG presenting to The Hospital for Sick Children. Their characteristics were: Genotype: H3K27M mutant, mean age 7.5 years, median age 7.1 years, 60% male.
Recruitment	We analysed samples from patients presenting to The Hospital for Sick Children with a diagnosis of H3K27M-mutant DIPG and who consented to use of their tissue for research purposes.
Ethics oversight	The Hospital for Sick Children Research Ethics Board

Note that full information on the approval of the study protocol must also be provided in the manuscript.
